# Distribution, Contents, and Types of Mycosporine-Like Amino Acids (MAAs) in Marine Macroalgae and a Database for MAAs Based on These Characteristics

**DOI:** 10.3390/md18010043

**Published:** 2020-01-07

**Authors:** Yingying Sun, Naisheng Zhang, Jing Zhou, Shasha Dong, Xin Zhang, Lei Guo, Ganlin Guo

**Affiliations:** 1 State Key Laboratory of Food Science and Technology, Jiangnan University, Wuxi 214122, China; zns371322@163.com (N.Z.); dongss34@163.com (S.D.); zhangxin@163.com (X.Z.); guol@jou.edu.cn (L.G.); 2 Jiangsu Key Laboratory of Marine Bioresources and Eco-Environment, Jiangsu Ocean University, Lianyungang 222005, China; guoganlin@163.com; 3 A Co-Innovation Center of Jiangsu Marine Bio-industry Technology, Lianyungang 222005, China; 4 Lianyungang of Products Quality Supervision and Inspection, Lianyungang 222006, China; zhoujing158@163.com

**Keywords:** CiteSpace analysis, marine macroalgae, mycosporine-like amino acids, online database for MAAs, photoproduction

## Abstract

Mycosporine-like amino acids (MAAs), maximally absorbed in the wavelength region of 310–360 nm, are widely distributed in algae, phytoplankton and microorganisms, as a class of possible multi-functional compounds. In this work, based on the Web of Science, Springer, Google Scholar, and China national knowledge infrastructure (CNKI), we have summarized and analyzed the studies related to MAAs in marine macroalgae over the past 30 years (1990–2019), mainly focused on MAAs distribution, contents, and types. It was confirmed that 572 species marine macroalgae contained MAAs, namely in 45 species of Chlorophytes, 41 species of Phaeophytes, and 486 species of Rhodophytes, and they respectively belonged to 28 orders. On this basis, we established an open online database to quickly retrieve MAAs in 501 species of marine macroalgae. Furthermore, research concerning MAAs in marine macroalgae were analyzed using CiteSpace. It could easily be seen that the preparation and purification of MAAs in marine macroalgae have not been intensively studied during the past 10 years, and therefore it is necessary to strengthen the research in the preparation and purification of MAA purified standards from marine macroalgae in the future. We agreed that this process is not only interesting, but important due to the potential use of MAAs as food and cosmetics, as well as within the medicine industry.

## 1. Introduction

MAAs (mycosporine-like amino acids) are natural compounds with low molecular weight and exist in diverse aquatic organisms, from algae [1,2,3,4,5,6,7] to phytoplankton [2,7,8,9,10,11,12], cyanobacteria [2,7,13,14,15], microorganisms [16,17], and marine animals [18,19,20,21]. 

MAAs have maxima absorption ranging between 310 and 360 nm [22]. They constitute a class of more than 30 related UV-absorbing compounds with molecules constituted by imino-carbonyl derivatives of mycosporine cyclohexenone chromophore [7,23]. MAAs exhibited the scavenging effects of oxygen free radicals [24,25], anti-lipid oxidation activity [26], a regulatory role in plant growth and development [27], and so on [28]. Meanwhile, MAAs may also possess many physiological functions, including the protection of antitumor activity [29], embryonic and larval development [30], reproduction regulation [23], and osmotic regulation [31].

MAAs were found in marine organisms in the 1960s [17]. However, research pertaining to MAAs in marine macroalgae gradually attracted the attention of researchers in the 1990s [32,33,34]. More exactly, most studies concerning MAAs in marine macroalgae focused on the period of 1998–2009 [2,4,5,7,35,36]. During this period, MAAs in more than 300 species of marine macroalgae were determined, for example, the green algae *Acrosiphonia arcta* [4], *Boodlea composite* [5], *Prasiola crispa* ssp. *antarctica* [3], *Prasiola crispa* [35] and others macroalgae [4,5,7] in Chlorophyta; a class of the brown algae in Phaeophyta, *Ecklonia radiata* [37], *Halopteris scoparia* [4], *Hydroclathrus clathratus* [5], *Laminaria saccharina* [38], and others brown algae [4,5,7]; many red algae of Rhodophyta, *Ahnfeltiopsis devoniensis* [39], *Apophlaea lyallii* [37], *Bangia atropurpurea* [40], *Chondrus crispus* [41], *Gracilaria chilensis* [42], *Iridaea* sp. [43], *Palmaria palmata* [44], *Porphyra umbilicalis* [1] and etc. [4,5,7,45,46]. From 2009 to 2019, MAAs in less than 200 species, such as *Bangia atropurpurea* [47], *Calliarthron tuberculosum* [48], *Catenella repens* [49], *Gelidium lingulatum* [50], *Gloiopeltis furcate* [51], *Palmaria palmata* [52], *Pyropia plicata* [53], *Solieria chordalis* [54] and others macroalgae [55,56,57,58], were found. 

Marine macroalgae as marine primary productivity have developed complex and unique metabolic pathways. Therefore, they have been become the target of exploiting natural bioactive components. To date, MAAs in more than 500 marine macroalgae have been reported. However, unfortunately, the summary and generalization about these researches were lacked. This made it very difficult to provide relevant information on MAAs of marine macroalgae. Therefore, this paper will provide an overview of MAAs in marine macroalgae since 1990 and focus on the contents and types of MAAs, and their distribution in marine macroalgae. The two databases related MAAs in marine macroalgae have been showed [2,59]. Although these two databases were not specific databases for MAAs in marine macroalgae, and provide less information regarding MAAs, the lack of total MAAs contents and specific MAA content, these works provided us with a good idea. Therefore, further in paper we established an open online database for MAAs in 501 species of marine macroalgae to quickly retrieve some related information regarding MAAs in marine microalgae since 1990. A lot of information, such as the source (field/culture) and collection site of marine macroalgae, the total MAAs contents, specific MAA content, and/or MAAs composition etc., were included in this online database (http://210.28.32.218/MAAs/). 

CiteSpace was a software used in scientific literature to identify and display new trends of scientific development [60]. Finally, in this review, the development tendency of the studies about MAAs in marine macroalgae will be analyzed using CiteSpace to point out future research directions for researchers in related fields. This is not only very interesting, but also can promote the development of MAAs in marine macroalgae. 

## 2. Results

### 2.1. MAAs Distribution, Contents and Types in Marine Macroalgae

**Figure 1:**
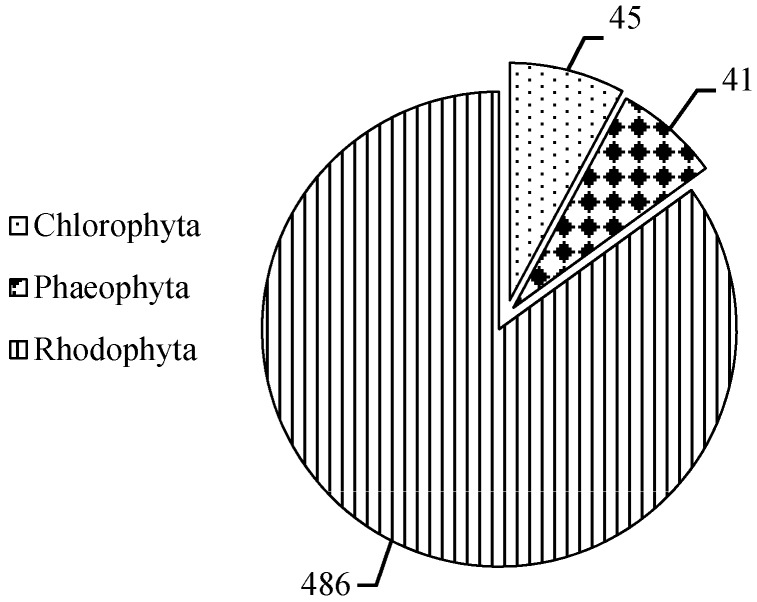
Distribution of MAAs in different phylums of marine macroalgae. Data extracted from related studies since 1990, and the number in a pie chart represents the total number of marine macroalgae species belong to this phylum.

**Figure 2:**
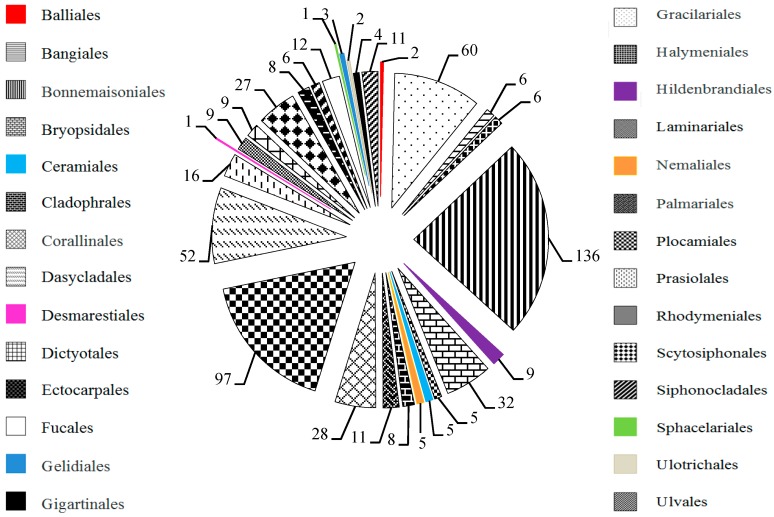
Distribution of MAAs in different orders of marine macroalgae. Data extracted from related studies since 1990, and the number in a pie chart represents the total number of marine macroalgae species belong to this order. The several highlights parts in the pie chart are just for clarity.

In our survey that summarized of more than 660 species, 572 species of marine macroalgae contained MAAs, found namely in 45 species in Chlorophyta, 41 species in Phaeophyta, and 486 species in Rhodophyta (Figure 1), and they respectively belonged to 28 orders (Figure 2). Among them, marine macroalgae which contained MAAs mainly distributed in orders Bangiales, Ceramiales, Gigartinales, and Gracilariales. MAAs rfepresent a class of the secondary metabolites in marine macroalgae [61,62], and in addition to antioxidant [24,25,26,27,28] and anti-ultraviolet radiation activities [22,23,63], likely possess many physiological activities, such as capacity to inhibit the proliferation of tumor cells [29], protecting embryonic and larval development [30], and regulating reproduction [23] and osmosis [31]. 

**Figure 3:**
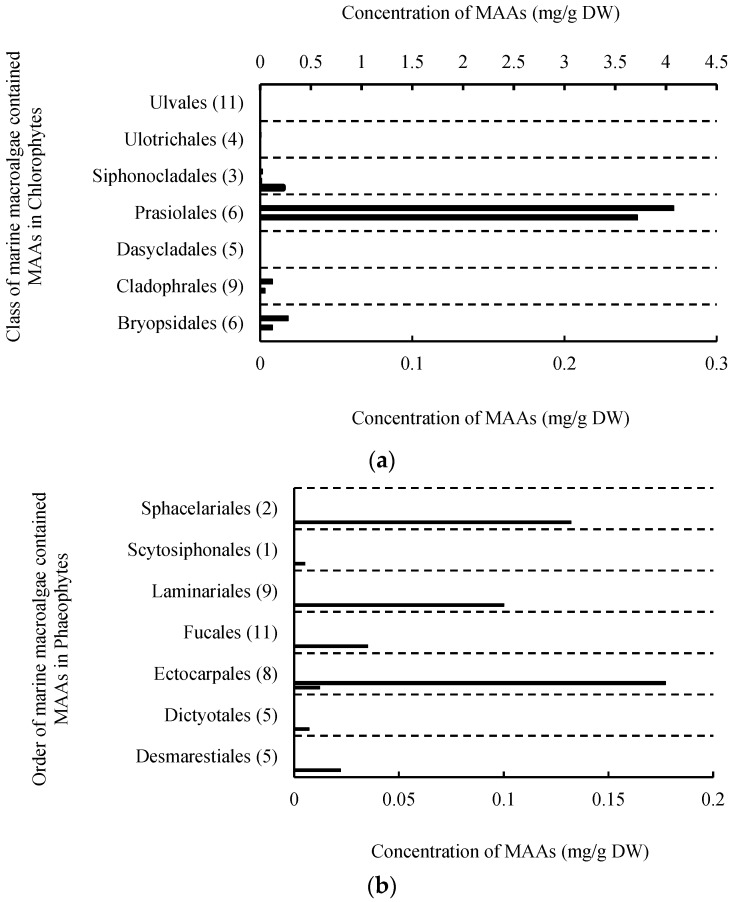

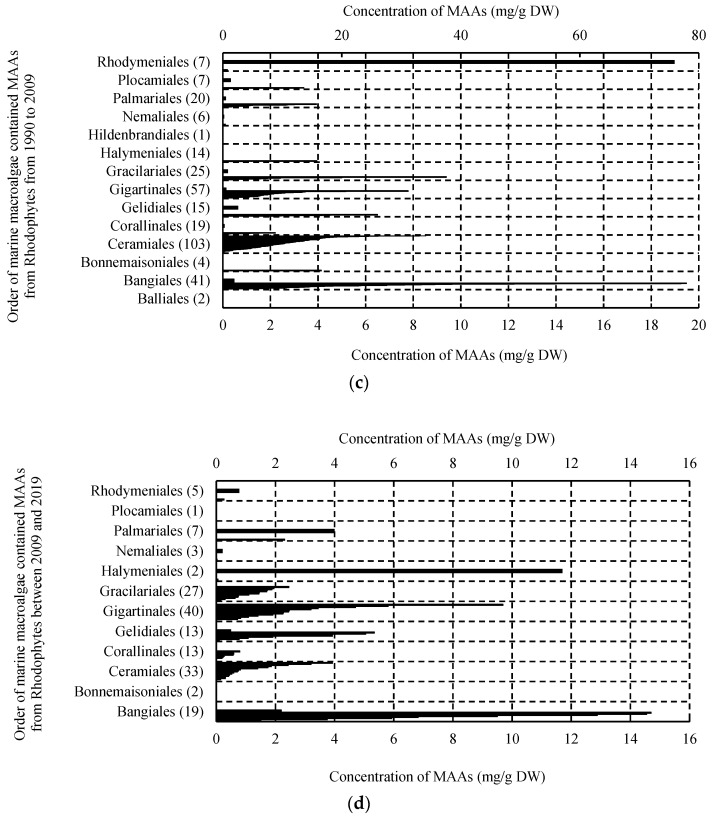
The contents of total MAAs in different orders of marine macroalgae. Data extracted from related studies since 1990. The number in bracket represents the number of species included in orders, bar represents a marine macroalgae with corresponding MAAs in (**a**–**d**), and the bars with different widths represent the numbers of some marine macroalgae with corresponding MAAs in (**c**,**d**).

**Figure 4:**
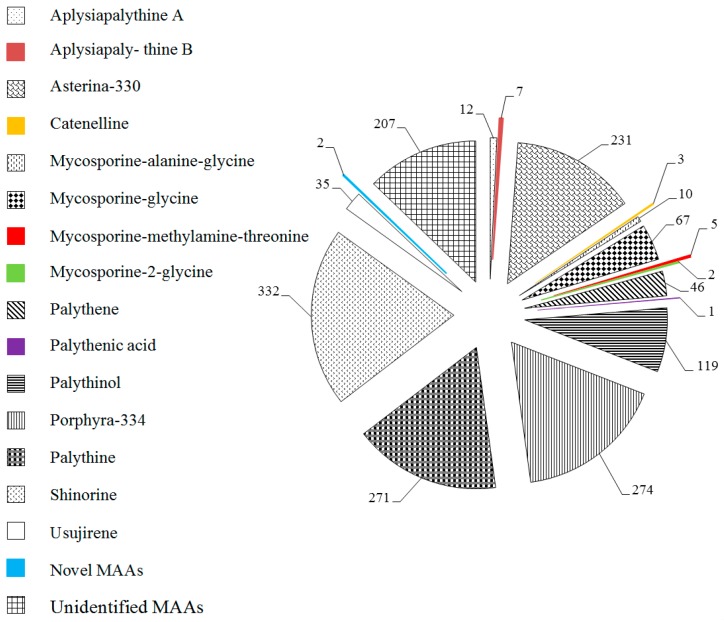
Types of MAAs in macroalgal Rhodophytes. Data extracted from related studies since 1990, and the number in a pie chart represents the total number of macroalgal Rhodophyta contained identical MAA. The several highlights parts in the pie chart are just for clarity.

The research efforts made pertaining to MAAs in marine macroalgae have drawn signifficant attention since 1990. The contents and composition of MAAs in marine macroalgae were important in developing MAAs. Therefore, the summary and analysis of total MAAs contents and MAAs composition in marine macroalgae have been conducted according to different orders in this paper (Figure 2, Figure 3 and Figure 4).

MAAs have been found in 45 species of the green algae in Chlorophyta [2,3,4,5,7,35,64]. Among them, total MAAs contents were determinied in only 12 species. The green algae with high total MAAs contents belong to Prasiolales, and contents were more than 3.5 mg/g DW (Figure 3a), such as *Prasiola crispa* ssp. *antarctica* [3], *Prasiola crispa* collected from South Shetlands [40] and Kongsfjorden [35] respectively. The total MAAs contents in other macroalgal Chlorophytes were very low, with values ranging between 0.0045 and 0.275 mg/g DW. Therefore, some bars which represent the total MAAs contents in different marine Chlorophytes were almost on the ordinate. 

The brown algae (41 species) in Phaeophyta also were confirmed to contain MAAs [2,3,4,5,32,37,38] (Table 1). Further, for eight species, namely *Chorda tomentosa* [4], *Desmarestia menziesii* [32], *Dictyota bartayresii* [5], *Dictyosiphon foeniculaceus* [4], *Pilayella littoralis* [4], *Ecklonia radiata* [37], *Halopteris scoparia* [4], *Hydroclathrus clathratus* [5] and *Sargassum oligocystum* [5], the total MAAs contents were pointed out. These values were very low and less than 0.2 mg/g DW (Figure 3b). 

A total of 486 MAA-containing strains of macroalgal Rhodophytes were detected. In order to present these reports from 1990 to 2019 more clearly, they are shown in two maps in Figure 3c,d, with 2009 as the cut-off time point. Among them, the total MAAs contents in 323 red algae were determined, and their MAAs contents were usually higher compared with macroalgal Chlorophytes and Phaeophytes [2,3,4,5,40,65]. For example, some members of orders Bangiales [5,40,65], Gracilariales [3,32,66], Gigartinales [5,40,42], and Gelidiales [48,67], their total MAAs contents ranges from 2 mg/g DW to nearly 20 mg/g DW. Of course, there were also many macroalgal Rhodophytes with lower total MAAs contents, such as *Actinotrichia fragilis* [5], *Asparagopsis taxiformi* [5], *Galaxaura oblongata* [5], *Gelidium corneum* [68], and *Georgiella confluens* [3], etc. [3,4,5,69], and these values were even less than 0.1 mg/g DW. Further, we classified these red algae into three groups according to their total MAAs contents. The first group, including 65 species belong to order Balliales, Ceramiales, Corallinales, Nemaliales and Rhodymeniales, exhibited low total MAAs contents (< 1 mg/g DW). A second group with the higher concentration of total MAAs and 52 species showed 1~2 mg/g DW MAAs. The last group that included 216 species, such as the orders Bangiales, Gelidiales, Gigartinales, and Gracilariales macroalgae showed the highest total MAAs contents and these values are all above 2 mg/g DW. Of note, *Rhodymenia* spp. Belonging to order Rhodymeniales had a surprisingly high MAAs contents (8.8–142.9 mg/g DW, average value 75.85 mg/g DW) [37]. This is clearly displayed in Figure 2c with another axe (maximum value of abscissa 80). In addition, the MAAs content value in each marine macroalgae could be obtained using the database (http://210.28.32.218/MAAs/) built by our team, which we will explain in detail later. 

From 1990 to 2019, the survey found that MAAs in marine macroalgae mainly focus on macroalgal Rhodophytes (Figure 1), therefore, MAAs types in Rhodophytes have been pointed out according to different orders of macroalgal Rhodophytes in this review (Figure 4). Identified MAAs in macroalgal Rhodophytes included 22 types, namely Aplysiapalythine A, Aplysiapalythine B, asterina-330, catenelline, mycosporine-alanine-glycine, mycosporine-glycine, mycosporine-methylamine-threonine, mycosporine-2-glycine, palythene, palythenic acid, palythinol, porphyra-334, palythine, shinorine, usujirene, and new MAAs prasiolin [70] and bostrychines A–F [71]. Among them, shinorine, porphyra-334, palythine and asterina-330 were more abundant, followed by palythinol; catenelline, mycosporine-2-glycine, mycosporine-methylamine-threonine and palythenic acid were very few. Among them, aplysiapalythine A, aplysiapaly thine B, catenelline, and novel MAAs (prasiolin and bostrychines A–F) were only found in macroalgal Rhodophytes [49,70,71,72]. Moreover, it is noted that MAAs have not yet been identified in many species of macroalgal Rhodophytes, and therefore it is quite possible that new MAAs will be discovered from those macroalgae. Seven MAAs, such as asterina-330, mycosporine-glycine, palythene, palythinol, porphyra-334, palythine, and shinorine were found in macroalgal Chlorophytes and Phaeophytes, however other types MAAs have not been found in them except for unidentified MAAs. These results can be queried through the database mentioned above.

**Figure 5:**
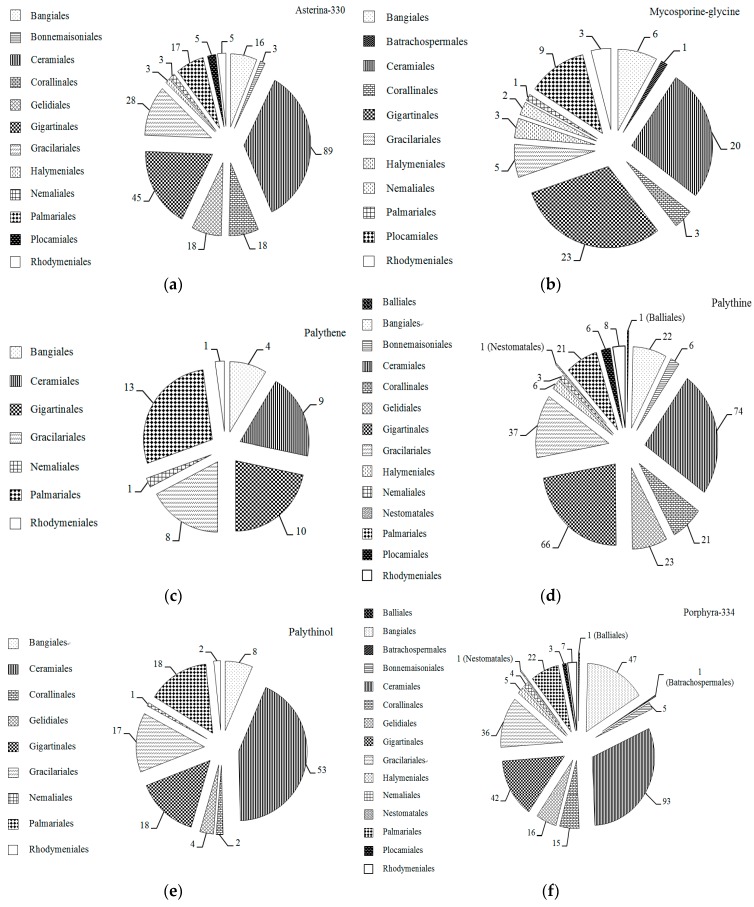

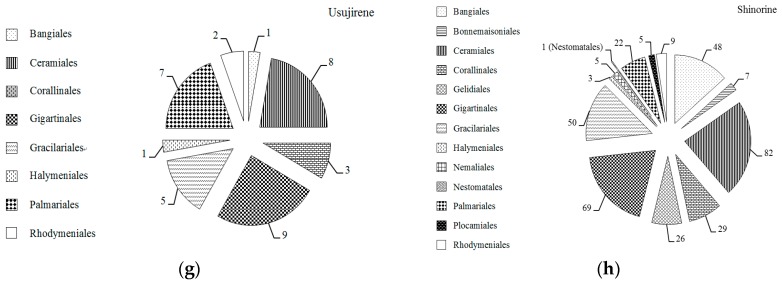
Distribution of specific MAA in different orders of macroalgal Rhodophytaes. Data extracted from related studies since 1990, and the number in a pie chart represents the total number of red macroalgae that contained specific MAA belong to this order. (**a**–**h**) represent specific MAA Asterina-330, Mycosporine-glycine, Palythene, Palythine, Palythinol, Porphyra-334, Usujirene and Shinorine, respectively.

In Figure 5, main MAAs in macroalgal Rhodophytes, such as asterina-330, mycosporine-glycine, palythene, palythinol, porphyra-334, palythine, shinorine and usujirene, were listed according to different orders. It can be clearly shown that these MAAs were common in marine macroalgae belonging to orders Ceramiales and Gigartinales macroalgae. Further, porphyra-334, palythine and shinorine were commonly found in orders Bangiales and Gracilariales macroalgae. And porphyra-334 and shinorine were also the most common in macroalgal Chlorophytes and Phaeophytes, but they usually occurred in lower concentrations. Meanwhile, mycosporine-glycine seemed to have the highest concentration in Chlorophytes and Phaeophytes species [4,5,73], for example, the proportion of mycosporine-glycine in MAAs for Chlorophytes *Boodlea composita* [5], *Chaetomorpha aerea* [4], *Codium fragile* [73], *Enteromorpha intestinalis* [73], *Halimeda polentia* [73], *Rhizoclonium tortuosum* [73], *Spongomorpha spinescens* [73], and *Ulva lactuca* [73] is more than 53%; in Phaeophytes *Agarum cribosum* [73], *Alaria esculenta* [73], *Ascophyllum nodosum* [73], *Chorda filum* [73], *Drsmarestia aculeata* [73], *Elachista fucicola* [73], *Fucus vesiculosis* [73], *Laminaria saccharina* [73], *Laminariocolas tomentosoides* [73], and *Sargassum fluitans* [73], this proportion was higher than 87%, even as high as 100% in *Alaria esculenta* [73], *Elachista fucicola* [73], *Fucus vesiculosis* [73], *Laminaria saccharina* [73], and *Laminariocolas tomentosoides* [73]. In Rhodophytes, such as *Acanthophora specifera* [5], *Chondrus crispus* [73], *Corallina officinalis* [73], *Cystoclonium purpureum* [73], *Lomentaria orcadensis* [73], *Mastocarpus stellata* [73], *Phycodrys rubens* [73], *Porphyra umbilicalis* [73], and other red algae [2,3,4,5,6,7,32,33,34,35,40,42,43,44,45,55,69,71,74,75,76,77,78], the proportion of mycosporine-glycine in MAAs was lower than 50%; and relatively numerous species (more than 260 species) did not detect this MAA, for instance, *Actinotrichia fragilis* [5], *Ahnfeltiopsis devoniensis* [39], *Amphiroa rigida* [2], *Asparagopsis armata* [4], and *Bangia atropurpurea* [3], etc. [1,2,3,4,5,40,41,42,44,55,58,65,67,68,69,70,71,72,74,76,79,80,81,82,83,84,85,86,87]. However, in Rhodophytes *Apophlaea lyallii* [37] and *Palmaria palmata* [73], the proportion of mycosporine-glycine in MAAs was very high. Therefore, those Chlorophytes [4,5,73], Phaeophytes [73], and two Rhodophytes [37,73] species mentioned above were good sources of mycosporine-glycine. Some reports have determined that mycosporine-glycine, porphyra-334, and shinorine have better antioxidants properties [29,39,88,89], and therefore many macroalgal Rhodophytes species which belonged to the orders Bangiales, Ceramiales, Gigartinales, and Gracilariales have been considered to constitute prolific sources of porphyra-334 and shinorine, e.g., *Acanthophora specifera* [5], *Bangia atropurpurea* [65], *Caloglossa apomeiotica* [5], *Porphyra dioica* [57], *Bostrychia radicans* [40], *Ceramium nodulosum* [4], *Catenella impudica* [40], *Curdiea racovitzae* [32], *Gracilaria domingensis* [90], *Gymnogongrus griffithsiae* [4], and *Mastocarpus stellatus* [4], etc. [3,5,32,40,48,50,74,85,91,92,93]. 

In addition, it is worth mentioning that the difference of the total MAAs or MAA value between cultivated marine macroalgae and field material existed from same collection location. For exsample, cultured *Stictosiphonia tangatensi* [40] exhibited only 47.8% of the total MAAs found in the field sample [5]. *Chondrus crispus*, which in culture esposured to green or blue light radiation, exhibited asterina-330, palythene, palythinol and shinorine that were lack of MAAs in the field sample. Similary phenomenons also occurred in other seaweeds, such as *Bostrychia radicans* [5,40], *Caloglossa stipitata* [5,49], *Chondrus crispus* [60,72], *Kallymenia antarctica* [3], *Mazzaella laminarioides* [74], *Neuroglossum ligulatum* [3], *Palmaria decipiens* [3], *Plocamium cartilagineum* [3], *Porphyra columbina* [6,43], and *Porphyra endiviifolium* [3]. In the database that we set up later, we made clear the source of marine macroalgae that was field or culture, or commercial provision.

The specific parameters of 10 types of MAAs in marine macroalgae, such as structure, extinction coefficient, retention time and maximum absorption wavelength, have been showed in Table 1. Unfortunately, some parameters of these MAAs have not been determined, for instance, the extinction coefficient of mycosporine-2-glycine and usujirene. This should be due to the lack of commercially available MAAs standards.

In order to clearly present the distribution of specific MAA in each marine macroalgae, based on the literature information from the Web of Science, Springer, Google Scholar, and CNKI, Table 2 and Table 3 are presenteed. In these two tables, all marine macroalgae were grouped according to the types of MAAs that they contained. 

### 2.2. An Open Database for MAAs in Marine Macroalgae

In order for the scientists in the field to have a more comprehensive and clearer understanding of MAAs in marine macroalgae, it was necessary to establish a corresponding database. Therefore, our project team established a database (http://210.28.32.218/MAAs/) of MAAs in marine macroalgae over the past nearly 30 years utilizing data information from the Web of Science, Springer, Google Scholar and CNKI. In this database, more detailed information in relation to algal MAAs, such as the total contents of MAAs, content of specific MAA, type of MAAs, origin of marine macroalgae, and/or composition of specific MAA in MAAs, was listed. This was the comprehensive summary database of MAAs in marine macroalgae at home and abroad, and it was open and free. 

Up to now, it has been determined that 572 species of marine macroalgae contained MAAs. Among them, MAAs contents and/or MAAs composition in 501 species have been reported. Therefore, related informations of MAAs in 501 species of marine macroalgae since 1990 have been getted using our database for MAAs in marine macroalgae. In the following work process, we will try to expand the sources of the reports collection and its published time in order to provide more complete data about MAAs in marine macroalgae. 

### 2.3. Marine Macroalgae with No Detectable MAAs Concentrations

**Figure 6:**
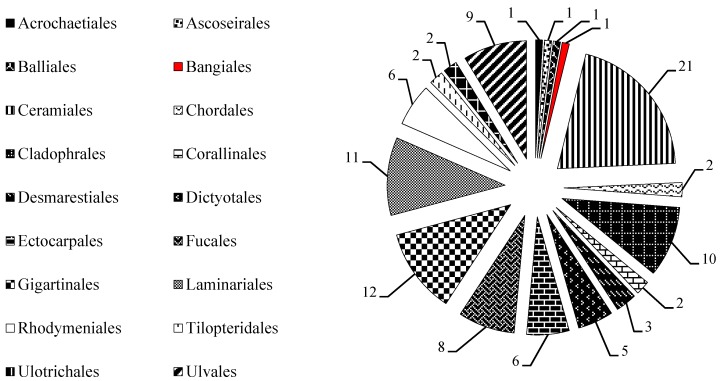
Distribution of marine macroalgae with no detectable MAAs concentrations. Data collected from related studies since 1990, and the number in a pie chart represents the number of marine macroalgae belonging to these orders.

MAAs accumulation in marine macroalgae were widespread and but not ubiquitous characteristics, and some marine macroalgae did not contain MAAs with detectable concentrations. We found that these marine macroalgae distributed in 18 orders (Figure 6) and its numbers were more than 100 species (Table 4) in the past 30 years. From Figure 6 it appeared that marine macroalgae with no detectable MAA concentrations have a wide taxonomic distribution. 

The green algae (21 species) in Chlorophyta, the brown algae (37 species) in Phaeophyta, and the red algae (45 species) in Rhodophyta were confirmed to have no detectable MAAs concentrations (Table 4), such as macroalgal Chlorophytes *Acrosiphonia arcta* [3], *Acrosiphonia penicilliformis* [3], *Anadyomene wrightii* [3], and etc. [3]; macroalgal Phaeophytes *Adenocystis utricularis* [3], *Alaria esculenta* [3], *Ascoseira mirabilis* [3], and others brown algae [3]; macroalgal Rhodophytes *Antarcticothamnion polysporum* [3], *Heterosiphonia plumosa* [70], *Odonthalia dentate* [4], and so on [3,70]. 

MAAs were an important class of bioactive secondary metabolites in marine macroalgae [67,68], their types and accumulation were variable with some environmental variables, including radiation [6,74,77,81,116], nutrients [6,74,77,81], salinity [44], temperature [116], and desiccation [113,117]. These studies were not included in this paper. 

### 2.4. Trends in Research on MAAs in Marine Macroalgae

**Figure 7:**
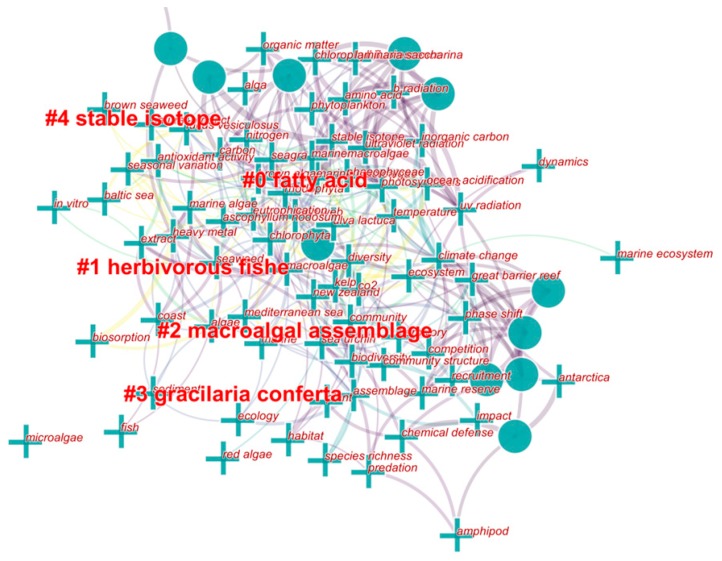
Cluster view of MAAs studies in marine algae between 2009 and 2019. Data extracted from related studies collected in Web of Science.

Figure 7 shows a cluster view of studies about MAAs in marine macroalgae in the past 10 years (2009–2019). Cluster analysis demonstrated that these works were still dispersive (these larger dots and crosses don’t overplay and combine) and did not form a very concentrated research direction. For example, these investigations mainly included effects of ultraviolet radiation, nitrogen, temperature and climate change on MAAs contents and composition, the physiological activity and seasonal variation of MAAs, MAAs profile and distribution, and so on. And five clusters formed (#0-#4) presented some researches about MAAs can cluster in these several areas. It was very clear that the preparation and purification of MAAs in marine macroalgae did not catch enough attention of researchers in these studies between 2009 and 2019. 

MAAs were multi-functional compounds, namely included UV-photoprotective activity [54], antioxidant properties [90,118], and other possible activities, such as anti-desiccant, protective agents against temperature variations [119], and etc. [23,27,28,29,30,31]. Therefore, MAAs could be widely used in food, cosmetics, and medicine in the future [96]. Note that in this application, it becomes very important to study the preparation and purification of MAAs.

In fact, the extraction of MAAs in marine macroalgae has been involved in many studies which were mainly concentrated on the distribution [64,69,92,95,114], profile [119], physiological activity [55,67,71,103,120,121,122,123], properties [56,81,92,124,125], chemical characterization [54] of MAAs and effects of some environment factors on MAAs [36,54,66,74,79,80,86,101,102,103,109,125,126]. However, further their isolation and purification did not conduct.

Until now, there were few studies on the isolation, purification, and preparation of MAAs in marine macroalgae. Six novel mycosporine-like amino acids, bostrychines A–F, were obtained from *Bostrychia scorpioides* [71]. A new MAA catenelline was isolated from *Catenella repens* [49]. MAAs in *Chondrus crispus* [57], *Palmaria palmata* [57], *Porphyra dioica* [57], *Porphyra haitanensis* [126] and *Rhodymenia pseudopalmata* [103] have been separated and identified. The preparation of porphyra-334 in *Bangia atropurpurea* [47], *Eucheuma* [127], *Gracilaria changii* [94], *Porphyra vietnamensis* [78], *Porphyra yezoensis* [128], and *Rhodymenia pseudopalmata* [103] has been reported. Isolation and purification of others MAAs in *Agarophyton chilense* [104], *Ahnfeltiopsis devoniensis* [39,129], *Bostrychia scorpioides* [76,77], *Champia novae-zelandiae* [104], *Chlamydomonas hedlyei* [123], *Chondracanthus chamissoi* [50], *Chondrus crispus* [41,57], *Gelidium corneum* [39,129], *Gelidium lingulatum* [50], *Gracilaria changii* [108,130], *Gracilaria cornea* [87], *Mastocarpus stellatus* [76,77], *Palmaria decipiens* [123], *Palmaria palmata* [57,131], *Porphyra dioica* [57], *Porphyra rosengurttii* [39,119], *Porphyra* sp. [32,131], *Porphyra tenera* [123,132], *Porphyra yezoensis* [76,77,105,128], *Pyropia leucosticte* [39] and *Pyropia plicata* [104], such as palythine, palythinol, shinorine, usujirene and *etc.*, that were often used as standards for identification and quantification of MAAs, also have been researched. The extraction process of MAAs in *Eucheuma* [124,133], *Gloiopeltis furcata* [51], *Gracilaria chilensis* [105], *Porphyra* sp., [134,135], *Porphyra haitanensis* [135] and *Porphyra yezoensis* [136] has been pointed. Therefore, the purification and preparation of MAAs in marine macroalgae need to be explored in future studies.

## 3. Materials and Methods 

### 3.1. Methods

Utilizing data obtained from the Web of Science, Springer, Google Scholar, and CNKI, the reliable material sources of this systematic manuscript paper were obtained from literature published during the last thirty years. Further, to provide more explicit knowledge, the development tendency of the studies about MAAs in marine macroalgae has been analyzed by using CiteSpace (4.0) [60]. Corresponding data on MAAs in marine macroalgae was extracted from related studies collected using the Web of Science during 2009 and 2019 years.

### 3.2. Total MAAs Contents and Specific MAA Concentration 

In this work, total MAAs content and/or specific MAA concentration in each marine macroalgae species was not clearly listed, however, they were obtained using an online database (http://210.28.32.218/MAAs/), which was built by our team. A great deal of reports which were collected from the Web of Science, Springer, Google Scholar, and CNKI were built for this database. 

## 4. Conclusions 

This review summed up the basic situation of MAAs in 572 species of marine macroalgae which belonged to 28 orders, from 1990 to 2019, in particular, 45 species in Chlorophyta, 41 species in Phaeophyta, and 486 species in Rhodophyta, and found the existence of 22 fully characterized MAAs and a large number of unidentified MAA(s) in them. Five MAAs, namely shinorine, porphyra-334, palythine, asterina-330, and palythinol were the most common in Rhodophytes, followed by mycosporine-glycine, palythene, and usujirene. Among them, seven MAAs, including asterina-330, mycosporine-glycine, palythene, palythinol, porphyra-334, palythine, and shinorine, were found in Chlorophytes and Phaeophytes. In addition, so far, aplysiapalythine A, aplysiapaly thine B, catenelline, prasiolin and bostrychines A–F only have been found in Rhodophytes. According to different orders of marine macroalgae, the total MAAs contents in 12 species macroalgal Chlorophytes, 8 species macroalgal Phaeophytes and 323 species macroalgal Rhodophytes were pointed out in this work. Meanwhile, we detailed the structure, extinction coefficient, retention time, and maximum absorption wavelength of 10 common MAAs. 

Further, an open online database (http://210.28.32.218/MAAs/) for MAAs in 501 species of marine macroalgae was established on datas metioned above to quickly retrieve information related to MAAs in marine macroalgae since 1990. In this database, the source (field/culture) and collection site of marine macroalgae, total MAAs content, MAA type and/or content have been listed. 

Finally, the studies about MAAs in marine macroalgae were analyzed using CiteSpace considering the past 10 years, and the result demonstrated that the purification and preparation of MAA purified standards from marine macroalgae constitute a domain worthy to be penetratingly explored in future studies. 

In order to gain better knowledge about the current states and progress of MAAs in marine macroalgae, more reports have to be collected regarding MAAs from other sources of data. Overall, based on data from the last 30 years, our work provided more a comprehensive reference and fast inquiry about MAAs in marine macroalgae for relevant researchers. 

## Figures and Tables

**Table 1:** Structure, molar extinction coefficient, retention time, and maximum absorption wavelength of MAAs in marine macroalgae [43,94].

MAA	Structure	Extinction Coefficient ε (M^−1^ cm^−1^)	Maximum Absorption Wavelength (nm)
Asterina-330	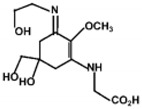	43800	330
Mycosporine-2-glycine	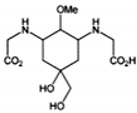	—	334
Mycosporine-glycine	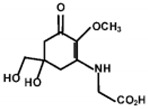	28100	310
Palythene	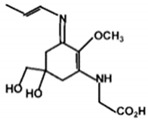	50000	360
Palythine	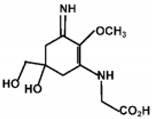	35500~36200	320
Palythenic acid	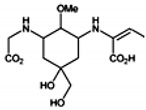	29200	337
Palythinol	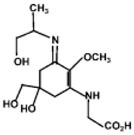	43500	332
Porphyra-334	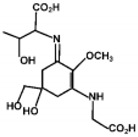	42300	334
Shinorine	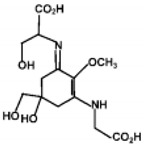	43700	334
Usujirene	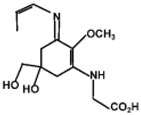	—	357

**Table 2:** MAAs in macroalgal Chlorophytes and Phaeophytes.

MAAs	Green Macroalgae	Ref.	Brown Macroalgae	Ref.
AS	*Codium adhaerens*, *Codium fragile*	[7]	*Padina crassa*	[5]
MG	*Boodlea composite*, *Chaetomorpha tortuosa*, *Codium adhaerens*, *Prasiola crispa* ssp. *antarctica*	[4,7,34]	*Desmarestia menziesii*	[5]
PE	*Caulerpa weberiana*, *Chaetomorpha tortuosa*	[7,34]		
PL	*Codium adhaerens*	[7]	*Padina crassa*	[5]
PR	*Acetabularia mediterranea*, *Acrosiphonia arcta, Acrosiphonia penicilliformis*, *Caulerpa racemosa*, *Cladophora rupestris*, *Codium adhaerens*, *Codium fragile*, *Dictyosphaeria cavernosa*, *Enteromorpha bulbosa*, *Ulva lactuca*	[2,4,5,7,75]	*Ascophyllum nodosum*, *Chorda tomentosa*, *Desmarestia aculeata*, *Desmarestia menziesii*, *Dictyosiphon foeniculaceus*, *Dictyota bartayresii*, *Halopteris scoparia*, *Himantothallus grandifolius*, *Hydroclathrus clathratus*, *Padina crassa*, *Pilayella littoralis*, *Sargassum oligocystum*	[3,4,5,7,34,75]
PI	*Acetabularia mediterranea*, *Cladophora rupestris*, *Codium adhaerens*, *Codium fragile*	[7,75]	*Desmarestia menziesii*, *Halopteris scoparia*, *Himantothallus grandifolius*, *Padina crassa*, *Sargassum oligocystum*	[3,4,5,7,48]
SH	*Acetabularia mediterranea*, *Caulerpa weberiana*, *Cladophora rupestris*, *Codium adhaerens*, *Codium fragile*, *Dictyosphaeria cavernosa*, *Monostroma hariotii*, *Valonia aegagropila*	[2,5,7,34,75]	*Ascophyllum nodosum*, *Desmarestia aculeata*, *Fucus spiralis*, *Halopteris scoparia*, *Padina crassa*, *Sargassum oligocystum*	[4,5,7,34,75]
UN	*Enteromorpha intestinalis*, *Prasiola stipata*, *Prasiola crispa* ssp. *antarctica*	[2,75]	*Prasiola crispa*	[49]

Note: AS, Asterina-330; MG, Mycosporine-glycine; PE, Palythene; PL, Palythinol; PR, Porphyra-334; PI, Palythine; SH, Shinorine; UN, Unidentified MAAs.

**Table 3:** MAAs in macroalgal Rhodophytes.

MAAs	Red Macroalgae	Ref.
APA	*Agarophyton chilense*, *Bostrychina arbuscula*^a^, *Ceramium* sp. ^a^, *Euptilota formosissima* ^a^, *Gigartina macrocarpa* ^a^, *Hymenena affinis*, *Mastocarpus stellatus* ^a^, *Porphyra umbilicalis* ^a^, *Pyropia columbina* ^a^, *Pyropia plicata* ^a^, *Rhodophyllis membranecea* ^a^, *Sarcothalia atropurpurea* ^a^, *Spongoclonium pastorale* ^a^	[71]
APB	*Agarophyton chilense*, *Champia novae-zelandiae*, *Gigartina macrocarpa*, *Porphyra umbilicalis*, *Pyropia columbina*, *Pyropia plicata*, *Sarcothalia atropurpurea*	[71]
AS	*Acanthophora muscoides, Acanthophora specifera*, *Agarophyton chilense*, *Amansia multifida*, *Ahnfeltiopsis devoniensis*, *Actinotrichia fragilis*, *Arthrocardia gardneri*, *Asparagopsis armata*, *Bangia atropurpurea*, *Bonnemaisonia hamifera*, *Bostrychia arbuscula*, *Bostrychia calliptera*, *Bostrychia montagnei*, *Bostrychia moritziana*, *Bostrychia radicans*, *Bostrychia scorpioides*, *Bostrychia simpliciuscula*, *Bryothamnion seaforthii*, *Bostrychia tenella*, *Bryothamnion triquetrum*, *Caloglossa apomeiotica*, *Caloglossa leprieurii*, *Calliarthron tuberculosum*, *Caloglossa stipitata*, *Caloglossa ogasawaraensis*, *Centroceras clavulatum*, *Ceramium nodulosum*, *Ceramium* sp., *Ceramium secundatum*, *Champia novae-zelandiae*, *Chondracanthus acicularis*, *Chondracanthus elegans*, *Chondracanthus teedei*, *Chondrus crispus*, *Chondrus ocellatus*, *Corallina officianalis* var. *chilensisa*, *Corallina officinalis*, *Corallina* sp., *Corallina vancouveriensis*, *Craspedocarpus erosus*, *Cryptonemia crenulata*, *Curdiea racovitzae*, *Devaleraea ramentacea*, *Dichotomaria marginata*, *Digenea simplex*, *Ellisolandia elongata*, *Euptilota formosissima*, *Gastroclonium ovatum*, *Gelidiella acerosa*, *Gelidiopsis variabilis*, *Gelidium amansii*, *Gelidium crinale*, *Gelidium corneum*, *Gelidium floridanum*, *Gelidium pusillum*, *Gelidium sesquipedale*, *Gloiopeltis furcata*, *Gigartina macrocarpa*, *Gigartina pistillata*, *Gigartina skottsbergii*, *Gracilaria caudata*, *Gracilaria changii*, *Gracilaria chilensis*, *Gracilaria cornea*, *Gracilaria conferta*, *Gracilaria eucheumoides*, *Gracilaria domingensis*, *Gracilaria saliconia*, *Gracilaria tenuistipitata*, *Gracilaria vermiculophylla*, *Gracilariopsis longissima*, *Gracilariopsis tenuifrons*, *Gymnogongrus antarctica*, *Gymnogongrus antarcticus*, *Gymnogongrus griffithsiae*, *Hypnea musciformis*, *Hypnea spinella*, *Iridaea chordata*, *Jania adhaerens*, *Jania crassa*, *Jania cubensis*, *Jania rubens*, *Jania subulata*, *Kallymenia antarctica*, *Laurencia caraibica*, *Laurencia cartilaginea*, *Laurencia changii*, *Laurencia dendroidea*, *Laurencia filiformis*, *Laurencia obtusa*, *Lithophyllum incrustans*, *Lithophyllum expansum*, *Mastocarpus stellatus*, *Mazzaella flaccida*, *Mazzaella laminarioides*, *Myriogramme manginii*, *Notophycus fimbriatus*, *Osmundea hybrid*, *Osmundea pinnatitida*, *Osmundea spectabilis*, *Pachymenia laciniata*, *Palmaria decipiens*, *Palmaria palmata*, *Palisada flagellifera*, *Palisada perforate*, *Phyllophora appendiculata*, *Plocamium cartilagineum*, *Polysiphonia arctica*, *Porphyra endiviifolium*, *Porphyra columbina*, *Porphyra leucosticta*, *Porphyra rosengurttii*, *Porphyra* ssp., *Prionitis lanceolata*, *Pterocladiella capillacea*, *Pyropia acanthophora*, *Pyropia columbina*, *Pyropia plicata*, *Rhodophyllis membranecea*, *Rhodymenia pseudopalmata*, *Sarcothalia atropurpurea*, *Solieria filiformis*, *Spongoclonium pastorale*, *Spyridia clavata*, *Stictosiphonia arbuscula*, *Stictosiphonia hookeri*, *Stictosiphonia intricate*, *Stictosiphonia tangatensis*, *Tricleocarpa cylindrica*, *Vertebrata lanosa*	[3,4,5,6,7,32,35,37,39,40,41,42,44,48,49,51,52,55,66,67,70,71,74,75,78,79,80,81,95,96,97,98,99,100,101,102,103,104]
CL	*Catenella caespitosa*, *Catenella repens*, *Catenella nipae*	[49,72]
MAG	*Champia novae-zelandiae, Ceramium* sp., *Gigartina macrocarpa*, *Mastocarpus stellatus*, *Porphyra umbilicalis*, *Pyropia columbina*, *Pyropia plicata*, *Rhodophyllis membranecea*, *Sarcothalia atropurpurea*, *Spongoclonium pastorale*	[78]
MG	*Acanthophora muscoides, Acanthophora specifera*, *Agarophyton chilense*, *Apophlaea lyallii*, *Blastophyllis calliblepharoides*, *Bostrychia moritziana*, *Bostrychia radicans*, *Bostrychia scorpioides*, *Centroceras clavulatum*, *Ceramium rubrum*, *Ceramium* sp., *Champia novae-zelandiae*, *Chondria arinata*, *Curdiea racovitzae*, *Devaleraea ramentacea*, *Dumontia incrassata* ^a^, *Gracilaria caudata*, *Gracilaria cornea*, *Grateloupia lanceola*, *Gymnogongrus turquetii*, *Hypnea spinella*, *Iridaea chordata*, *Jania subulata*, *Kallymenia antarctica*, *Laurencia caraibica*, *Laminaria saccharina*, *Mazzaella laminarioides*, *Notophycus fimbriatus*, *Osmundaria obtusiloba*, *Pachymenia orbicularis*, *Palmaria decipiens*, *Palmaria palmata*, *Phyllophora antarctica*, *Phyllophora appendiculata*, *Porphyra columbina*, *Porphyra endiviifolium*, *Porphyra purpurea-violacea*, *Pyropia plicata*, *Rhodymenia* spp., *Sarcothalia atropurpurea*, *Schizymenia apoda*, *Spongoclonium pastorale*	[2,3,4,5,6,7,32,34,35,37,40,42,43,44,45,55,69,71,74,75,76,77,78]
MMT	*Agarophyton chilense*, *Ceramium* sp., *Porphyra umbilicalis*, *Pyropia columbina*, *Pyropia plicata*, *Sarcothalia atropurpurea*	[77]
M2G	*Gloiopeltis furcata*	[40]
PE	*Acanthophora specifera*, *Actinotrichia fragilis*, *Agarophyton chilense*, *Bangia atropurpurea*, *Bostrychia simpliciuscula*, *Ceramium nodulosum*, *Chondrus crispus*, *Curdiea racovitzae*, *Devaleraea ramentacea*, *Gigartina macrocarpa*, *Gracilaria changii*, *Gracilaria chilensis*, *Gracilaria tenuistipitata*, *Gracilariopsis tenuifrons*, *Gracilaria vermiculophylla*, *Iridaea chordata*, *Osmundea hybrid*, *Osmundea pinnatitida*, *Palmaria decipiens*, *Palmaria palmata*, *Phyllophora antarctica, Phyllophora appendiculata*, *Porphyra purpurea-violacea*, *Pyropia plicata*, *Rhodymenia pseudopalmata*, *Sarcothalia atropurpurea*, *Spongoclonium pastorale*, *Stictosiphonia hookeri*, *Vertebrata lanosa*	[3,4,5,32,34,40,41,42,43,44,70,75,77,96,99,102,104]
PA	*Solieria chordalis*	[54]
PL	*Acanthophora specifera*, *Actinotrichia fragilis*, *Bangia atropurpurea*, *Bostrychia calliptera*, *Bostrychia montagnei*, *Bostrychia moritziana*, *Bostrychia radicans*, *Bostrychia simpliciuscula*, *Bostrychia tenella*, *Caloglossa leprieurii*, *Chondria arinata*, *Chondrus crispus*, *Corallina vancouveriensis*, *Curdiea racovitzae*, *Devaleraea ramentacea*, *Ellisolandia elongata*, *Gelidium corneum*, *Gelidium pusillum*, *Gelidium sesquipedale*, *Gracilaria changii*, *Gracilaria chilensis*, *Gracilaria cornea*, *Gracilaria domingensis*, *Gracilaria eucheumoides*, *Gracilaria saliconia*, *Gracilaria tenuistipitata*, *Gracilariopsis longissima*, *Gracilariopsis tenuifrons*, *Halopythis incurve*, *Iridaea chordata*, *Kallymenia antarctica*, *Laurencia cartilaginea*, *Laurencia changii*, *Laurencia obtusa*, *Mazzaella flaccida*, *Mastocarpus stellatus*, *Osmundea spectabilis*, *Palmaria decipiens*, *Palmaria palmata*, *Plocamium cartilagineum*, *Polysiphonia arctica*, *Porphyra endiviifolium*, *Rhodymenia pseudopalmata*, *Rhodymenia* spp., *Stictosiphonia intricate*, *Stictosiphonia tangatensis*	[3,4,5,32,35,37,39,40,41,42,44,45,48,52,55,65,67,75,79,96,97,98,99,102,104,105]
PR	*Acanthophora muscoides, Acanthophora specifera*, *Actinotrichia fragilis*, *Agarophyton chilense*, *Amansia multifida*, *Arthrocardia gardneri*, *Asparagopsis armata*, *Asparagopsis taxiformis*, *Bangia atropurpurea*, *Bangia fuscopurpurea*, *Blastophyllis calliblepharoides*, *Bostrychia arbuscula*, *Bostrychia calliptera*, *Bostrychia harveyi*, *Bostrychia montagnei*, *Bostrychia moritziana*, *Bostrychia radicans*, *Bostrychia scorpioides*, *Bostrychia simpliciuscula*, *Bostrychia tenella*, *Bryothamnion seaforthii*, *Calliarthron tuberculosum*, *Caloglossa apomeiotica*, *Caloglossa leprieurii*, *Caloglossa ogasawaraensis*, *Caloglossa stipitata*, *Calliarthron tuberculosum*, *Catenella nipae*, *Ceramium nodulosum*, *Ceramium* sp., *Champia novae-zelandiae*, *Chondracanthus acicularis*, *Chondria arinata*, *Chondrus crispus*, *Corallina officinalis*, *Corallina officianalis* var. *chilensisa*, *Corallina vancouveriensis*, *Craspedocarpus erosus*, *Curdiea racovitzae*, *Cystoclonium purpureum*, *Devaleraea ramentacea*, *Dumontia incrassata*, *Endocladia muricata*, *Euptilota formosissima*, *Galaxaura oblongata*, *Ganonema farinosa*, *Gastroclonium ovatum*, *Gelidiella acerosa*, *Gelidiopsis variabilis*, *Gelidium amansii*, *Gelidium crinale*, *Gelidium corneum*, *Gelidium floridanum*, *Gelidium pusillum*, *Gelidium sesquipedale*, *Georgiella confluens*, *Gigartina macrocarpa*, *Gigartina skottsbergii*, *Gloiopeltis furcata*, *Gracilaria birdiae*, *Gracilaria caudata*, *Gracilaria changii*, *Gracilaria chilensis*, *Gracilaria conferta*, *Gracilaria cornea*, *Gracilaria domingensis*, *Gracilaria eucheumoides*, *Gracilaria saliconia*, *Gracilaria vermiculophylla*, *Gracilaria tenuistipitata*, *Grateloupia lanceola*, *Gymnogongrus griffithsiae*, *Halopythis incurve*, *Hymenena affinis*, *Hydropuntia cornea*, *Hypnea musciformis*, *Hypnea spinella*, *Iridaea* sp., *Iridaea chordata*, *Jania adhaerens*, *Jania rubens*, *Kallymenia antarctica*, *Laurencia caraibica*, *Laurencia cartilaginea*, *Laurencia changii*, *Laurencia dendroidea*, *Laurencia filiformis*, *Laurencia obtusa*, *Lithophyllum expansum*, *Lithophyllum incrustans*, *Lithothamnion antarcticum*, *Lithophyllum expansum*, *Mazzaella flaccida*, *Mastocarpus jardinii*, *Mastocarpus papillatus*, *Mastocarpus stellatus*, *Myriogramme manginii*, *Neuroglossum ligulatum*, *Nodularia spumigena*, *Notophycus fimbriatus*, *Osmundea hybrid*, *Osmundaria obtusiloba*, *Osmundea spectabilis*, *Pachymenia laciniata*, *Pachymenia orbicularis*, *Palmaria decipiens*, *Palmaria palmata*, *Palisada flagellifera*, *Palisada perforate*, *Pantoneura plocamioides*, *Plocamium cartilagineum*, *Phyllophora antarctica*, *Polysiphonia arctica*, *Porphyra dioica*, *Porphyra endiviifolium*, *Porphyra leucosticta*, *Porphyra plocamiestris*, *Porphyra purpurea*, *Porphyra purpurea-violacea*, *Porphyra rosengurttii*, *Porphyra* sp., *Porphyra tenera*, *Porphyra umbilicalis*, *Porphyra yezoensis*, *Porphyra vietnamensis*, *Prionitis lanceolata*, *Pseudolithophyllum expansum*, *Pterocladiella capillacea*, *Pterocladia* sp., *Ptilota plumosa*, *Pyropia acanthophora*, *Pyropia columbina*, *Pyropia plicata*, *Rhodophyllis membranecea*, *Rhodymenia pseudopalmata*, *Rhodymenia* spp., *Rhodymenia subantarctica*, *Sarcothalia atropurpurea*, *Schizymenia apoda*, *Scinaia boergesenii*, *Spongoclonium pastorale*, *Spyridia clavata*, *Spyridia filamentosa*, *Stictosiphonia arbuscula*, *Stictosiphonia hookeri*, *Stictosiphonia intricate*, *Stictosiphonia tangatensis*, *Tricleocarpa cylindrical*, *Vertebrata lanosa*	[1,3,4,5,6,7,32,34,35,37,40,41,42,44,45,47,48,51,52,55,57,58,65,66,67,69,71,74,75,76,77,78,79,84,85,86,87,88,90,91,92,95,100,102,104,105,106,107]
PI	*Acanthophora muscoides, Acanthophora specifera*, *Agarophyton chilense*, *Amansia multifida*, *Amphiroa rigida*, *Ahnfeltiopsis devoniensis*, *Arthrocardia gardneri*, *Asparagopsis armata*, *Asparagopsis taxiformis*, *Bangia atropurpurea*, *Blastophyllis calliblepharoides*, *Bonnemaisonia hamifera*, *Bostrychia arbuscula*, *Bostrychia calliptera*, *Bostrychia harveyi*, *Bostrychia moritziana*, *Bostrychia montagnei*, *Bostrychia pinnata*, *Bostrychia radicans*, *Bostrychia scorpioides*, *Bryothamnion seaforthii*, *Bostrychia tenella*, *Bryothamnion triquetrum*, *Calliarthron tuberculosum*, *Caloglossa ogasawaraensis*, *Centroceras clavulatum*, *Ceramium nodulosum*, *Ceramium secundatum*, *Ceramium* sp., *Champia novae-zelandiae*, *Chondracanthus acicularis*, *Chondracanthus chamissoi*, *Chondracanthus elegans*, *Chondracanthus teedei*, *Chondria arinata*, *Chondrus crispus*, *Chondrus ocellatus*, *Chondrus yendoi*, *Corallina elongata*, *Corallina officinalis*, *Corallina officianalis* var. *chilensisa*, *Corallina* sp., *Corallina vancouveriensis*, *Craspedocarpus erosus*, *Cryptonemia crenulata*, *Curdiea racovitzae*, *Devaleraea ramentacea*, *Dichotomaria marginata*, *Digenea simplex*, *Dumontia incrassata* ^a^, *Ellisolandia elongata*, *Endocladia muricata*, *Euptilota formosissima*, *Gastroclonium ovatum*, *Gelidiella acerosa*, *Gelidiopsis variabilis*, *Gelidium amansii*, *Gelidium corneum*, *Gelidium crinale*, *Gelidium floridanum*, *Gelidium lingulatum*, *Gelidium pusillum*, *Gelidium sesquipedale*, *Georgiella confluens*, *Gigartina macrocarpa*, *Gigartina pistillata*, *Gigartina skottsbergii*, *Gracilaria asiatica*, *Gracilaria birdiae*, *Gracilaria caudata*, *Gracilaria changii*, *Gracilaria chilensis*, *Gracilaria conferta*, *Gracilaria cornea*, *Gracilaria domingensis*, *Gracilaria eucheumoides*, *Gracilaria saliconia*, *Gracilaria tenuistipitata*, *Gracilaria vermiculophylla*, *Gracilariopsis longissima*, *Grateloupia lanceola*, *Gymnogongrus Antarctica*, *Gymnogongrus antarcticus*, *Gymnogongrus griffithsiae*, *Halopythis incurve*, *Hymenena affinis*, *Hypnea musciformis*, *Hypnea spinella*, *Hypoglossum hypoglossoides*, *Iridaea chordata*, *Iridaea* sp., *Jania adhaerens*, *Jania crassa*, *Jania cubensis*, *Jania rubens*, *Jania subulata*, *Kallymenia antarctica*, *Laurencia caraibica*, *Laurencia cartilaginea*, *Laurencia changii*, *Laurencia dendroidea*, *Laurencia filiformis*, *Laurencia obtusa*, *Lithophyllum incrustans*, *Lithophyllum expansum*, *Lithothamnion antarcticum*, *Lomentaria articulate*, *Mastocarpus jardinii*, *Mastocarpus stellatus*, *Mazzaella flaccida*, *Mazzaella laminarioides*, *Myriogramme manginii*, *Neuroglossum ligulatum*, *Notophycus fimbriatus*, *Osmundea hybrid*, *Osmundaria obtusiloba*, *Osmundea pinnatitida*, *Osmundea spectabilis*, *Pachymenia laciniata*, *Pachymenia orbicularis*, *Palmaria decipiens*, *Palmaria palmata*, *Palisada flagellifera*, *Palisada perforate*, *Phyllophora antarctica*, *Phyllophora appendiculata*, *Plocamium cartilagineum*, *Polysiphonia arctica*, *Polysiphonia urceolata*, *Porphyra leucosticta*, *Porphyra purpurea-violacea*, *Porphyra rosengurttii*, *Porphyra* sp., *Porphyra yezoensis*, *Porphyra umbilicalis*, *Prionitis lanceolata*, *Pterocladiella capillacea*, *Pterocladia* sp., *Pyropia columbina*, *Pyropia acanthophora*, *Pyropia plicata*, *Rhodophyllis membranecea*, *Rhodymenia pseudopalmata*, *Rhodymenia* spp., *Sarcothalia atropurpurea*, *Sarcothalia papillosa*, *Schizymenia apoda*, *Scinaia boergesenii*, *Solieria filiformis*, *Solieria pacifica*, *Spongoclonium pastorale*, *Stictosiphonia arbuscula*, *Stictosiphonia intricate*, *Stictosiphonia tangatensis*, *Tricleocarpa cylindrical*, *Vertebrata lanosa*	[3,4,5,6,7,32,34,35,36,37,39,40,41,42,43,44,45,46,48,50,52,55,56,58,65,66,67,68,69,70,71,75,76,77,78,79,82,83,84,90,91,95,97,100,101,102,108,109,110]
SH	*Acanthophora muscoides*, *Acanthophora spicifera*, *Actinotrichia fragilis*, *Agarophyton chilense*, *Ahnfeltiopsis devoniensis*, *Amansia multifida*, *Amphiroa rigida*, *Amphiroa* sp., *Ahnfeltiopsis devoniensis*, *Arthrocardia gardneri*, *Asparagopsis armata*, *Asparagopsis taxiformis*, *Bangia atropurpurea, Bangia fuscopurpurea*, *Bangia* sp., *Bonnemaisonia hamifera*, *Bostrychina arbuscula*, *Bostrychia calliptera*, *Bostrychia moritziana*, *Bostrychia montagnei*, *Bostrychia moritziana*, *Bostrychia scorpioides*, *Bostrychia simpliciuscula*, *Bostrychia tenella*, *Bryothamnion seaforthii*, *Bryothamnion triquetrum*, *Callithamnion tetragonum*, *Caloglossa apomeiotica*, *Caloglossa leprieurii*, *Caloglossa ogasawaraensis*, *Caloglossa stipitata*, *Calliarthron tuberculosum*, *Centroceras clavulatum*, *Ceramium echionotum*, *Ceramium nodulosum, Ceramium rubrum*, *Ceramium secundatum*, *Ceramium* sp., *Champia novae-zelandiae*, *Chondracanthus acicularis*, *Chondracanthus chamissoi*, *Chondracanthus elegans*, *Chondracanthus teedei*, *Chondria arinata*, *Chondrus crispus*, *Chondrus yendoi*, *Corallina elongata*, *Corallina officinalis*, *Corallina officianalis* var. *chilensisa*, *Corallina* sp., *Corallina vancouveriensis*, *Craspedocarpus erosus*, *Cryptonemia crenulata*, *Curdiea racovitzae*, *Cystoclonium purpureum* ^a^, *Devaleraea ramentacea*, *Dichotomaria marginata*, *Digenea simplex*, *Dumontia incrassata* ^a^, *Ellisolandia elongata*, *Endocladia muricata*, *Euptilota formosissima*, *Galaxaura oblongata*, *Ganonema farinosa*, *Gastroclonium ovatum*, *Gelidiella acerosa*, *Gelidiopsis variabilis*, *Gelidium amansii*, *Gelidium corneum*, *Gelidium crinale*, *Gelidium floridanum*, *Gelidium latifolium*, *Gelidium lingulatum*, *Gelidium pusillum*, *Gelidium* sp., *Gelidium sesquipedale*, *Georgiella confluens*, *Gloiopeltis furcata*, *Gigartina macrocarpa*, *Gigartina pistillata*, *Gigartina skottsbergii*, *Gracilaria birdiae*, *Gracilaria caudata*, *Gracilaria cornea*, *Gracilaria changii*, *Gracilaria chilensis*, *Gracilaria conferta*, *Gracilaria cornea*, *Gracilaria domingensis*, *Gracilaria eucheumoides*, *Gracilaria gracilis*, *Gracilaria saliconia*, *Gracilaria tenuistipitata*, *Gracilaria vermiculophylla*, *Gracilariopsis longissima*, *Grateloupia doryphora*, *Gymnogongrus Antarctica*, *Gymnogongrus antarcticus*, *Gymnogongrus griffithsiae*, *Gymnogongrus turquetii*, *Halopythis incurve*, *Hypnea musciformis*, *Hypnea spinella*, *Hypoglossum hypoglossoides*, *Iridaea chordata*, *Iridaea* sp., *Jania adhaerens*, *Jania crassa*, *Jania cubensis*, *Jania rubens*, *Jania subulata*, *Kallymenia antarctica*, *Laurencia caraibica*, *Laurencia changii*, *Laurencia dendroidea*, *Laurencia filiformis*, *Laurencia obtusa*, *Lithophyllum expansum*, *Lithophyllum incrustans*, *Lithophyllum expansum*, *Lithothamnion antarcticum*, *Lomentaria articulate*, *Mazzaella flaccida*, *Mazzaella laminarioides*, *Mastocarpus jardinii*, *Mastocarpus papillatus*, *Mastocarpus stellatus*, *Myriogramme manginii*, *Neuroglossum ligulatum*, *Notophycus fimbriatus*, *Osmundaria obtusiloba*, *Osmundea pinnatitida*, *Osmundea spectabilis*, *Pachymenia laciniata*, *Palmaria decipiens*, *Palmaria palmata*, *Palisada flagellifera*, *Palisada perforate*, *Pantoneura plocamioides*, *Phyllophora antarctica*, *Phyllophora appendiculata*, *Plocamium cartilagineum*, *Polysiphonia arctica*, *Porphyra dioica*, *Porphyra endiviifolium*, *Polysiphonia urceolata*, *Porphyra haitanensis*, *Porphyra leucosticta*, *Porphyra plocamiestris*, *Porphyra purpurea*, *Porphyra rosengurttii*, *Porphyra* sp., *Porphyra umbilicalis*, *Porphyra yezoensis*, *Prionitis lanceolata*, *Pseudolithophyllum expansum* ^a^, *Pterocladia lucida*, *Pterocladiella capillacea*, *Pterocladia* sp., *Pyropia acanthophora*, *Pyropia columbina*, *Pyropia plicata*, *Rhodomela virgata*, *Rhodophyllis membranecea*, *Rhodymenia pseudopalmata*, *Rhodymenia* spp., *Rissoella verruculosa*, *Sarcothalia atropurpurea*, *Sarcothalia papillosa*, *Schizymenia apoda*, *Scinaia boergesenii*, *Spongoclonium pastorale*, *Spyridia clavata*, *Stictosiphonia arbuscula*, *Stictosiphonia hookeri*, *Stictosiphonia intricate*, *Stictosiphonia tangatensis*, *Trichocarpus crinitus*, *Tricleocarpa cylindrica*	[1,3,4,5,6,7,32,34,35,36,37,38,39,40,42,43,44,45,48,50,51,52,53,55,56,57,66,67,68,70,71,75,76,77,78,79,82,84,85,86,90,91,92,96,98,101,103,105,108,110,111,112,113]
US	*Agarophyton chilense*, *Bostrychia scorpioides*, *Calliarthron tuberculosum*, *Ceramium nodulosum*, *Ceramium* sp., *Chondracanthus acicularis*, *Corallina officianalis* var. *chilensisa*, *Corallina vancouveriensis*, *Gigartina macrocarpa*, *Gracilariopsis tenuifrons*, *Gracilaria vermiculophylla*, *Mastocarpus papillatus*, *Mastocarpus stellatus*, *Mazzaella flaccida*, *Osmundea hybrid*, *Osmundea pinnatitida*, *Palmaria decipiens*, *Palmaria palmata*, *Phyllophora Antarctica*, *Prionitis lanceolata*, *Pterocladiella capillacea*, *Pyropia plicata*, *Rhodophyllis membranecea*, *Rhodymenia pseudopalmata*, *Sarcothalia atropurpurea*, *Spongoclonium pastorale*, *Vertebrata lanosa*	[4,6,37,39,52,55,70,76,77,78,104]
NM	*Bostrychia scorpioides*, *Gracilaria vermiculophylla*	[70,71]
UN	*Ahnfeltiopsis devoniensis* I~II, *Asparagopsis armata*, *Bonnemaisonia hamifera*, *Bostrychia calliptera*, *Bostrychia scorpioides* I~II, *Bostrychia* sp., *Callithamnion tetragonum*, *Callophyllis variegata*, *Catenella caespitosa* I~II, *Catenella nipae* I~IX, *Catenella impudica*, *Catenella repens* I~II, *Ceramium nodulosum* I~II, *Ceraminum rubrum*, *Ceramium secundatum* I~II, *Chondracanthus acicularis* I~II, *Chondria arinata*, *Chondrus crispus* I~II, *Chondrus ocellatus*, *Corallina officinalis*, *Corallina* sp. I~II, *Curdiea racovitzae* I~II, *Devaleraea ramentacea*, *Gastroclonium ovatum*, *Gelidium corneum* I~II, *Gigartina pistillata* I~II, *Gigartina skottsbergii*, *Gigartina skottsbergii*, *Gracilaria changii*, *Gracilaria conferta*, *Gracilaria gracilis*, *Gracilaria lemaneiformis* I~II, *Gracilaria vermiculophylla* I~III, *Gracilariopsis longissima*, *Gracilariopsis tenuifrons*, *Hypoglossum hypoglossoides*, *Iridaea chordata*, *Iridaea tuberculosa*, *Jridaea cordata*, *Kallymenia antarctica*, *Lophurella hoockeriana*, *Mazzaella laminarioides*, *Mastocarpus stellatus* I~II, *Myriogramme manginii*, *Neuroglossum ligulatum*, *Nothogenia fastigiata*, *Notophycus fimbriatus*, *Nothogenia* sp., *Osmundea hybrid*, *Osmundea pinnatitida*, *Palmaria decipiens*, *Palmaria palmata* I~III, *Phyllophora truncata*, *Polysiphonia arctica*, *Polysiphonia* sp., *Porphyra dioica*, *Porphyra endiviifolium*, *Porphyra rosengurttii*, *Porphyria* spec., *Porphyra yezoensis*, *Pseudolithophyllum expansum*, *Ptilota gunneri, Ptilonia magellanica*, *Pyropia columbina*, *Rhodomela confervoides*, *Sarcothalia crispata*, *Sarcothalia papillosa*, *Schizoseris* sp., *Solieria chordalis*, *Vertebrata lanosa*	[1,3,4,6,7,37,40,44,47,54,57,69,70,75,86,88,90,102,105,114,115]

Note: APA, Aplysiapalythine A [^a^. Tentative identification]; APB, Aplysiapalythine B; AS, Asterina-330; CL, Catenelline; MAG, mycosporine-alanine-glycine; MG, Mycosporine-glycine; MMT, mycosporine-methylamine-threonine; M2G, Mycosporine-2-glycine; PE, Palythene; PA, Palythenic acid; PI, Palythine; PL, Palythinol; PR, Porphyra-334; PS, Palythine-serine; SH, Shinorine; SME, Shinorine methyl ester; US, Usujirene; NM, Novel MAAs; UN, Unidentified MAAs.

**Table 4:** Marine macroalgae with no detectable MAAs concentrations.

	Marine Macroalgae
**Chlorophyta**	*Acrosiphonia arcta* [3], *Acrosiphonia penicilliformis* [3], *Anadyomene wrightii* [3], *Boergesenia forbesii* [3], *Chaetomorpha linum* [3], *Chaetomorpha melagonium* [3], *Cladophora rupestris* [3], *Enteromorpha compressa* [3], *Enteromorpha intestinalis* [3], *Enteromorpha* sp. [3], *Enteromorpha* spp. [3], *Monostroma nitidum* [3], *Monostoma arcticum* [3], *Ulva conglohata* [3], *Ulva fasciata* [3], *Ulva lactuca* [3], *Ulva olivascens* [3], *Ulva rotundata* [3], *Valoniopsis pachynema* [3]
**Phaeophyta**	*Adenocystis utricularis* [3], *Alaria esculenta* [3], *Ascoseira mirabilis* [3], *Chnoospora implexa* [3], *Chorda filum* [3], *Chordaria flagelliformis* [3], *Colpomenia sinuosa* [3], *Cystoseira usneoides* [3], *Desmarestia aculeata* [3], *Desmarestia menziesii* [3], *Dictyota dichotoma* [3], *Fucus distichus* [3], *Fucus serratus* [3], *Fucus spiralis* [3], *Fucus vesiculosus* [3], *Kjellmaniella crassifolia* [3], *Laminaria digitata* [3], *Laminaria hyperborean* [3], *Laminaria japonica* [3], *Laminaria ochroleuca* [3], *Laminaria saccharina* [3], *Laminaria solidungula* [3], *Padina boryana* [3], *Padina pavonica* [3], *Phaeurus antarcticus* [3], *Saccorhiza dermatodea* [3], *Saccorhiza polyschides* [3], *Sargassum muticum* [3]
**Rhodophyta**	*Antarcticothamnion polysporum* [3], *Audouinella purpurea* [3], *Ballia callitricha* [3], *Bornetia secundiflora* [70], *Calliblepharis jubata* [70], *Callithamnion tetragonum* [70], *Champia parvula* [70], *Chylocladia verticillata* [70], *Delesseria lancifolia* [3], *Delesseria sanguinea* [3], *Dilsea carnosa* [76], *Furcellaria lumbricalis* [3,76], *Griffithsia corallinoides* [70], *Hymenocladiopsis crustigena* [3], *Heterosiphonia plumosa* [70], *Membranoptera alata* [70], *Metacallophyllis laciniata* [70], *Myriogramme smithii* [3], *Odonthalia dentate* [4], *Pantoneura plocamioides* [3], *Phyllophora ahnfeltioides* [3], *Phycodrys austrogeorgica* [3], *Phycodrys rubens* [3,71], *Phycodrys quercifolia* [3], *Phyllophora truncata* [3], *Picconiella plumosa* [3], *Plocamium cartilagineum* [3], *Plumaria plumosa* [70], *Porphyra plocamiestris* [3], *Ptilota serrate* [3], *Polysiphonia elongata* [3], *Polyides rotundus* [3], *Sphaerococcus coronopifolius* [70]

## References

[1] Gröniger A., Hallier C., Häder D.P. (1999). Influence of UV radiation and visible light on *Porphyra umbilicalis*: Photoinhibition and MAA concentration. J. Appl. Phycol..

[2] Gröniger A., Sinha R.P., Klish M., Häder D.P. (2000). Photoprotective compounds in cyanobacteria, phytoplankton and macroalgae-a database. J. Photochem. Photobiol. B Biol..

[3] Hoyer K., Karsten U., Sawall T., Wiencke C. (2000). Photoprotective substances in Antarctic macroalgae and their variation with respect to depth distribution, different tissues and developmental stages. Mar. Ecol. Prog. Ser..

[4] Karsten U., Sawall T., Hanelt D., Bischof K., Figueroa F.L., Flores-Moya A., Wiencke C. (1998). An inventory of UV-absorbing mycosporine-like amino acids in macroalgae from polar to warm-temperate regions. Bot. Mar..

[5] Karsten U., Sawall T., Wiencke C. (1998). A survey of the distribution of UV-absorbing substances in tropical macroalgae. Phycol. Res..

[6] Korbee Peinado N., Abdala Díaz R.T., Figueroa F.L., Helbling E.W. (2004). Ammonium and UV radiation stimulate the accumulation of mycosporine-like amino acids in *Porphyra columbina* (Rhodophyta) from Patagonia, Argentina. J. Phycol..

[7] Sinha R.P., Klisch M., Groniger A., Hader D.P. (1998). Ultraviolet-absorbing/screening substances in cyanobacteria, phytoplankton and macroalgae. J. Photochem. Photobiol. B Biol..

[8] Balskus E.P., Walsh C.T. (2010). The genetic and molecular basis for sunscreen biosynthesis in cyanobacteria. Science.

[9] Carreto J.I., Carignan M.O., Daleo G., De Marco S.G. (1990). Occurrence of mycosporine-like amino acids in the red-tide dinoflagellate *Alexandrium excavatum*: UV photoprotective compounds?. J. Plankton Res..

[10] Hannach G., Sigleo A.C. (1998). Photoinduction of UV-absorbing compounds in six species of marine phytoplankton. Mar. Ecol. Prog. Ser..

[11] Vernet M., Neori A., Haxo F.T. (1989). Spectral properties and photosynthetic action in red-tide populations of Prorocentrum micans and Gonyaulax polyedra. Mar. Biol..

[12] Yentsch C.S., Yentsch C.M., Calkins J. (1982). The Attenuation of light by marine phytoplankton with specific reference to the absorption of near-UV radiation. The Role of Solar Ultraviolet Radiation in Marine Ecosystems.

[13] Garcia-Pichel F., Castenholz R.W. (1993). Occurrence of UV-absorbing, mycosporine-like compounds among cyanobacterial isolates and an estimate of their screening capacity. Appl. Environ. Microbiol..

[14] Karsten U., Garcia-Pichel F. (1996). Carotenoids and mycosporine-like amino acid compounds in members of the genus Microcoleus (Cyanobacteria): A chemosystematic study. Syst. Appl. Microbiol..

[15] Queseda A., Vincent W.F. (1997). Strategies of adaptation by Antarctic cyanobacteria to ultraviolet radiation. Eur. J. Phycol..

[16] Arai T., Nishijima M., Adachi K., Sano H. (1992). Isolation and Structure of a UV Absorbing Substance from the Marine Bacterium Micrococcus sp. AK-334.

[17] Favre-Bonvin J., Aprin N., Brevard C. (1976). Structure of mycosporine. Chemistry.

[18] Banaszak A.T., Trench R.K. (1995). Effects of ultraviolet (UV) radiation on marine microalgal–invertebrate symbioses. Ⅱ. The synthesis of mycosporine-like amino acids in response to exposure to UV in *Anthopleura elegantissima* and *Cassiopeia xamachana*. J. Exp. Mar. Biol. Ecol..

[19] Shick J.M., Dunlap W.C., Chalker B.E., Banaszak A.T., Rosenzweig T.K. (1992). Survey of ultraviolet radiation-absorbing mycosporine-like amino acids in organs of coral reef holothuroids. Mar. Ecol. Prog. Ser..

[20] Stochaj W.R., Dunlap W.C., Shick J.M. (1994). Two new UV-absorbing mycosporine-like amino acids from the sea anem-one Anthopleura elegantissima and the effects of zooxanthellae and spectral irradiance on chemical composition and content. Mar. Biol..

[21] Whitehead K., Karentz D., Hedges J.I. (2001). Mycosporine-like amino acids (MAAs) in phytoplankton, a herbivorous pteropod (*Limacina helicina*), and its pteropod predator (*Clione antarctica*) in McMurdo Bay, Antarctica. Mar. Biol..

[22] Cockell C.S., Knowland J. (1999). Ultraviolet radiation screening compounds. Biol. Rev..

[23] Bandarangyake W.M. (1998). Mycosporines: Are they nature’s sunscreens?. Nat. Prod. Rep..

[24] Gao X., He Q.M., Zhang Z.H., Xu J.C., Zhang L. (2010). Study on antioxidant activity of mycosporine-like amino acids in two seaweeds. Mar. Environ. Sci..

[25] Xu Z.H. (2010). The Preparation Techniques of Mycosporine-Like Amino Acid from *Porphyra Yezoensis*. Master’s Thesis.

[26] Nakayama R., Tamura Y., Kikuzaki H., Nakatani N. (1999). Antioxidant effect of the constituent of susabinori (*Porphyra yezoensis*). J. Am. Oil Chem. Soc..

[27] Maekawa Y. (1995). Application of UV absorbance of algae. Ocean.

[28] Oren A., Gunde-Cimerman N. (2007). Mycosporines and mycosproine-like amino acids: UV protectants or multipurpose secondary metabolites?. FEMS Microbiol. Lett..

[29] Dunlap W.C., Yamamoto Y. (1995). Small molecule antioxidants in marine organisms: Antioxidant activity of mycosporine-glycine. Comp. Biochem. Physiol. B Biochem. Mol. Biol..

[30] Adams N.L., Shick J.M. (1996). Mycosporine-like amino acids provide protection against ultraviolet radiation in eggs of the green sea urchin *Strongylocentrotus droebachiensis*. Photochem. Photobiol..

[31] Oren A. (1997). Mycosporine-like amino acids as osmotic solutes in a community of halophilic cyanobactera. Geomicrobiol. J..

[32] Karentz D., McEuen F.S., Land M.C., Dunlap W.C. (1991). Survey of mycosporine-like amino acid compounds in Antarctic marine organisms: Potential protection from ultraviolet exposure. Mar. Biol..

[33] Maegawa M., Kunieda M., Kida W. (1993). The influence of ultraviolet radiation on the photosynthetic activity of several algae from different depths. Jpn. J. Phycol..

[34] McClintock J.B., Karentz D. (1997). Mycosporine-like amino acids in 38 species of subtidal marine organisms from McMurdo Sound, Antarctica. Antarct. Sci..

[35] Karsten U., Escoubeyrou K., Charles F. (2009). The effect of re-dissolution solvents and HPLC columns on the analysis of mycosporine-like amino acids in the eulittoral macroalgae *Prasiola crispa* and *Porphyra umbilicalis*. Helgol. Mar. Res..

[36] Zacher K., Roleda M.Y., Wulff A., Hanelt D., Wiencke C. (2009). Responses of Antarctic *Iridaea cordata* (Rhodophyta) tetraspores exposed to ultraviolet radiation. Phycol. Res..

[37] Lamare M.D., Lesser M.P., Barker M.F., Barry T.M., Schimanski K.B. (2004). Variation in sunscreen compounds (mycosporine-like amino acids) for marine species along a gradient of ultraviolet radiation transmission within doubtful sound, New Zealand. N. Z. J. Mar. Freshw. Res..

[38] Apprill A.M., Lesser M.P. (2003). Effects of ultraviolet radiation on *Laminaria saccharina* in relation to depth and tidal height in the Gulf of Maine. Mar. Ecol. Prog. Ser..

[39] De la Coba F., Aguilera J., Figueroa F.L., Gálvez M.V., Herrera E. (2009). Antioxidant activity of mycosporine-like amino acids isolated from three red macroalgae and one marine lichen. J. Appl. Phycol..

[40] Karsten U., Sawall T., West J., Wiencke C. (2000). Ultraviolet sunscreen compounds in epiphytic red algae from mangroves. Hydrobiologia.

[41] Karsten U., Franklin L.A., Lüning K., Wiencke C. (1998). Natural ultraviolet radiation and photosynthetically active radiation induce formation of mycosporine-like amino acids in the marine macroalga *Chondrus crispus* (Rhodophyta). Planta.

[42] Huovinen P., Gómez I., Figueroa F.L., Ulloa N., Morales V., Lovengreen C. (2004). Ultraviolet-absorbing mycosporine-like amino acids in red macroalgae from Chile. Bot. Mar..

[43] Carreto J.I., Carignan M.O., Montoya N.G. (2005). A high-resolution reverse-phase liquid chromatography method for the analysis of mycosporine-like amino acids (MAAs) in marine organisms. Mar. Biol..

[44] Karsten U., Dummermuth A., Hoyer K., Wiencke C. (2003). Interactive effects of ultraviolet radiation and salinity on the ecophysiology of two Arctic red algae from shallow waters. Polar Biol..

[45] Karsten U., Bischof K., Hanelt D., Tüg H., Wiencke C. (1999). The effect of ultraviolet radiation on photosynthesis and ultraviolet-absorbing substances in the endemic Arctic macroalga *Devaleraea ramentacea* (Rhodophyta). Physiol. Plant..

[46] Karsten U. (2000). Occurrence of photoprotective mycosporine-like amino acid compounds (MAAs) in marine red macroalgae from temperate Australian wates. Proc.-Linn. Soc. N. S. W..

[47] Chuang L.F., Chou H.N., Sung P.J. (2014). Porphyra-334 isolated from the marine algae *Bangia atropurpurea*: Conformational performance for energy conversion. Mar. Drugs.

[48] Gravem S.A., Adams N.L. (2012). Sex and microhabitat influence the uptake and allocation of mycosporine-like amino acids to tissues in the purple sea urchin, *Strongylocentrotus purpuratus*. Mar. Biol..

[49] Hartmann A., Becker K., Karsten U., Remias D., Ganzera M. (2015). Analysis of mycosporine-like amino acids in selected algae and cyanobacteria by hydrophilic interaction liquid chromatography and a novel MAAs from the red alga *Catenella repens*. Mar. Drugs.

[50] Véliz K., Chandía N., Karsten U., Lara C., Thiel M. (2019). Geographic variation in biochemical and physiological traits of the red seaweeds *Chondracanthus chamissoi* and *Gelidium lingulatum* from the south east Pacific coast. J. Appl. Phycol..

[51] Zhang W. (2016). Study on Extraction, Antioxidation and Moisturizing Activities of MAAs from *Gloiopeltis furcata*. Master’s Thesis.

[52] Yuan Y.V., Westcott N.D., Hu C., Kitt D.D. (2009). Mycosporine-like amino acid composition of the edible red alga, *Palmaria palmata* (dulse) harvested from the west and east coasts of Grand Manan Island, New Brunswick. Food Chem..

[53] Diehl N., Michalik D., Zuccarello G.C., Karsten U. (2019). Stress metabolite pattern in the eulittoral red alga *Pyropia plicata* (Bangiales) in New Zealand-mycosporine-like amino acids and heterosides. J. Exp. Mar. Biol. Ecol..

[54] Bedoux G., Hardouin K., Marty C., Taupin L., Vandanjon L., Bourgougnon N. (2014). Chemical characterization and photoprotective activity measurement of extraxts from the red macroalgae *Solieria chordalis*. Bot. Mar..

[55] Athukorala Y., Trang S., Kwok C., Yuan Y.V. (2016). Antiproliferative and antioxidant activities and mycosporine-Like amino acid profiles of wild-harvested and cultivated edible Canadian marine red macroalgae. Molecules.

[56] Celis-Plá P.S.M., Martínez B., Quintano E., García-Sánchez M., Pedersen A., Navarro N.P., Copertino M.S., Mangaiyarkarasi N., Mariath R., Figueroa F.L. (2014). Short-term ecophysiological and biochemical responses of *Cystoseira tamariscifolia* and *Ellisolandia elongata* to environmental changes. Aquat. Biol..

[57] Guihéneuf F., Gietl A., Stengel D.B. (2018). Temporal and spatial variability of mycosporine-like amino acids and pigments in three edible red seaweeds from western Ireland. J. Appl. Phycol..

[58] Roleda M.Y., Zacher K., Wulff A., Hanelt D., Wiencke C. (2008). Susceptibility of spores of different ploidy levels from Antarctic *Gigartina skottsbergii* (Gigartinales, Rhodophyta) to ultraviolet radiation. Phycologia.

[59] Sinha R.P., Singh S.P., Häder D.P. (2007). Database on mycosporines and mycosporine-like amino acids (MAAs) in fungi, cyanobacteria, macroalgae, phytoplankton and animals. J. Photochem. Photobiol. B Biol..

[60] Chen C.M., Chen Y., Hou J.H., Liang Y.X. (2009). CiteSpace II: Detecting and visualizing emerging trends and transient patterns in scientific literature. J. China Soc. Sci. Tech. Inf..

[61] Häder D.-P., Kumar H.D., Smith R.C., Worrest R.C. (2007). Effects of solar UV radiation on aquatic ecosystems and interactions with climate change. Photochem. Photobiol. Sci..

[62] Rastogi R.P., Sinha R.P., Singh S.P., Häder D.-P. (2010). Photoprotective compounds from marine organisms. J. Ind. Microbiol. Biotechnol..

[63] Llewellyn C.A., Airs R.L. (2010). Distribution and abundance of MAAs in 33 species of microalgae across 13 classes. Mar. Drugs.

[64] Karsten U., Friedl T., Schumann R., Hoyer K., Lembcke S. (2005). Mycosporine-like amino acids and phylogenies in green algae: Prasiola and its relatives from the Trebouxiophyceae (Chlorophyta). J. Phycol..

[65] Boedeker C., Karsten U. (2005). The occurrence of mycosporine-like amino acids in the gametophytic and sporophytic stages of *Bangia* (Bangiales, Rhodophyta). Phycologia.

[66] Figueroa F.L., Israel A., Neori A., Martínez B., Malta E.J., Put A., Inken S., Marquardt R., Abdala-Díaz R., Korbee N. (2010). Effect of nutrient supply on photosynthesis and pigmentation to short-term stress (UV radiation) in *Gracilaria conferta* (Rhodophyta). Mar. Pollut. Bull..

[67] Álvarez-Gómez F., Korbee N., Figueroa F.L. (2016). Analysis of antioxidant capacity and bioactive compounds in marine macroalgal and lichenic extracts using different solvents and evaluation methods. Cienc. Mar..

[68] Quintano E., Celis-PláP S.M., Martínez B., Díez I., Muguerza N., Figueroa F.L., Gorostiaga J.M. (2019). Ecophysiological responses of a threatened red alga to increased irradiance in an in situ transplant experiment. Mar. Environ. Res..

[69] Briani B., Sissini M.N., Lucena L.A., Batista M.B., Costa L.O., Nunes J.M.C., Schmitz C., Ramlov F., Maraschin M., Korbee N. (2018). The influence of environmental features in the content of mycosporine-like amino acids in red marine algae along the Brazilian coast. J. Phycol..

[70] Lalegerie F., Lajili S., Bedoux G., Taupin L., Stiger-Pouvreau V., Connan S. (2019). Photo-protective compounds in red macroalgae from Brittany: Considerable diversity in mycosporine-like amino acids (MAAs). Mar. Environ. Res..

[71] Orfanoudaki M., Hartmann A., Miladinovic H., Ngoc H.N., Karsten U., Ganzera M. (2019). Bostrychines A–F, six novel mycosporine-like amino-acids and a novel betaine from the red alga *Bostrychia scorpioides*. Mar. Drugs.

[72] Franklin L., Kräbs G., Kuhlenkamp R. (2001). Blue light and UV-A radiation control the synthesis of mycosporine-like amino acids in *Chondrus crispus* (Florideophyceae). J. Phycol..

[73] Banaszak A.T., Lesser M.P., Kuffner I.B., Ondrusek M. (1998). Relationship between ultraviolet (UV) and mycosporine-like amino acids (MAAs) in marine organisms. Bull. Mar. Sci..

[74] Navarro N.P., Mansilla A., Figueroa F., Korbee N., Jofre J., Plastino E. (2014). Short-term effects of solar UV radiation and NO_3_^−^ supply on the accumulation of mycosporine-like amino acids in *Pyropia columbina* (Bangiales, Rhodophyta) under spring ozone depletion in the sub-Antarctic region, Chile. Bot. Mar..

[75] Aguilera J., Bischof K., Karsten U., Hanelt D. (2002). Seasonal variation in ecophysiological patterns in macroalgae from an Arctic fjord. II. Pigment accumulation and biochemical defence systems against high light stress. Mar. Biol..

[76] Korbee N., Huovinen P., Figueroa F.L., Aguilera J., Karsten U. (2005). Availability of ammonium influences photosynthesis and the accumulation of mycosporine-like amino acids in two *Porphyra* species (Bangiales, Rhodophyta). Mar. Biol..

[77] Huovinen P., Matos J., Sousa-Pinto I., Figueroa F.L. (2006). The role of nitrogen in photoprotection against high irradiance in the Mediterranean red alga *Grateloupia lanceola*. Aquat. Bot..

[78] Bhatia S., Sharma K., Namdeo A.G., Chaugule B., Kavale M., Nanda S. (2010). Broad-spectrum sun-protective action of Porphyra-334 derived from *Porphyra vietnamensis*. Pharmacogn. Res..

[79] Kräbs G., Bischof K., Hanelt D., Karsten U., Wiencke C. (2002). Wavelength-dependent induction of UV-absorbing mycosporine-like amino acids in the red alga *Chondrus crispus* under natural solar radiation. J. Exp. Mar. Biol. Ecol..

[80] Torres P.B., Chow F., Ferreira M.J.P., dos Santos D.Y.A.C. (2016). Mycosporine-like amino acids from *Gracilariopsis tenuifrons* (Gracilariales, Rhodophyta) and its variation under high light. J. Appl. Phycol..

[81] Barufi J.B., Korbee N., Oliveira M.C., Figueroa F.L. (2011). Effects of N supply on the accumulation of photosynthetic pigments and photoprotectors in *Gracilaria tenuistipitata* (Rhodophyta) cultured under UV radiation. J. Appl. Phycol..

[82] Gómez I., Figueroa F.L., Huovinen P., Ulloa N., Morales V. (2005). Photosynthesis of the red alga *Gracilaria chilensis* under natural solar radiation in an estuary in southern Chile. Aquaculture.

[83] Karsten U., West J.A. (2000). Living in the intertidal zone-seasonal effects on heterosides and sun-screen compounds in the red alga *Bangia atropurpurea* (Bangiales). J. Exp. Mar. Biol. Ecol..

[84] Cardozo K.H.M., Marques L.G., Carvalho V.M., Carignan M.O., Pinto E., Marinho-Soriano E., Colepicolo P. (2011). Analyses of photoprotective compounds in red algae from the Brazilian coast. Braz. J. Pharmacogn..

[85] Daniel S., Cornelia S., Fred Z. (2004). UV-A sunscreen from red algae for protection against premature skin aging. Cosmetic and Toiletries Manufacture worldwide. Food Chem..

[86] Figueroa F.L., Escassi L., Perez-Rodriguez E., Korbee N., Giles A.D., Johnsen G. (2003). Effects of short-term irradiation on photoinhibition and accumulation of mycosporine-like amino acids in sun and shade species of the red algal genus Porphyra. J. Photochem. Photobiol. B Biol..

[87] Sinha R.P., Klisch M., Almut G., Häder D.-P. (2000). Mycosporine-like amino acids in the marine red alga *Gracilaria cornea*-effects of UV and heat. Environ. Exp. Bot..

[88] De la Coba F., Aguilera J., de Gálvez M.V., Alvarez M., Gallego E., Figueroa F.L., Herrera E. (2009). Prevention of the ultraviolet effects on clinical and histopathological changes, as well as the heat shock protein-70 expression in mouse skin by topical application of algal UV-absorbing compounds. J. Dermatol. Sci..

[89] Berthon J.Y., Nachat-Kappes R., Bey M., Cadoret J.P., Renimela I., Filaire E. (2017). Marine algae as attractive source to skin care. Free Radic. Res..

[90] Torres P., Santos J.P., Chow F., Ferreira M.J.P., dos Santos D.Y.A.C. (2018). Comparative analysis of in vitro antioxidant capacities of mycosporine-like amino acids (MAAs). Algal Res..

[91] Figueroa F.L., Korbee N., Abdala-Díaz R., Jerez C.G., López de la Torre M., Güenaga L., Larrubia M.A., Gómez-Pinchetti J.L. (2012). Biofiltration of fishpond effluents and accumulation of N-compounds (phycobiliproteins and mycosporine-like amino acids) versus compounds (polysaccharides) in *Hydropuntia cornea* (Rhodophyta). Mar. Pollut. Bull..

[92] Hartmann A., Murauer A., Ganzera M. (2017). Quantitative analysis of mycosporine-like amino acids in marine algae by capillary electrophoresis with diode-array detection. J. Pharm. Biomed. Anal..

[93] Bischof K., Kräbs G., Hanelt D., Wiencke C. (2000). Photosynthetic characteristics and mycosporine-like amino acids under UVradiation: A competitive advantage of *Mastocarpus stellatus* over *Chondrus crispus* at the Helgoland shoreline?. Helgol. Mar. Res..

[94] Ito S., Hirata Y. (1977). Isolation and structure of a mycosporine from the zoanthid *Palythoa tuberculosa*. Tetrahedron Lett..

[95] Barceló-Villalobos M., Figueroa F.L., Korbee N., Álvarez-Gómez F., Abreu M.H. (2017). Production of mycosporine-like amino acids from *Gracilaria vermiculophylla* (Rhodophyta) cultured through one year in an integrated multi-trophic aquaculture (IMTA) system. Mar. Biotechnol..

[96] Carreto J., Carignan M.O. (2011). Mycosporine-like amino acids: Relevant secondary metabolites. Chemical and ecological aspects. Mar. Drugs.

[97] Cardozo K.H.M., Carvalho V.M., Pinto E., Colepicolo P. (2006). Fragmentation of mycosporine-like amino acids by hydrogen/deuterium exchange and electrospray ionisation tandem mass spectrometry. Rapid Commun. Mass Spectrom..

[98] Figueroa F.L., Barufi J.B., Malta E.J., Conde-Álvarez R., Nitschke U., Arenas F., Mata M., Connan S., Abreu M.H., Marquardt R. (2014). *Cystoseira tamariscifolia* (Heterokontophyta), *Ulva rigida* (Chlorophyta) and *Ellisolandia elongata* (Rhodophyta). Aquat. Biol..

[99] Hoyer K., Karsten U., Wiencke C. (2002). Induction of sunscreen compounds in Antarctic macroalgae by different radiation conditions. Mar. Biol..

[100] Ju Q., Tang X.X., Zhao X.W., Ren X.Q., Li Y.F. (2011). Effects of UV-B radiation and different light repair conditions on the early development of the tetraspores of *Chondrus ocellatus* Holm. Acta Oceanol. Sin..

[101] Ju Q., Xiao H., Wang Y., Tang X.X. (2015). Effects of UV-B radiation on tetraspores of *Chondrus ocellatus* Holm (Rhodophyta), and effects of red and blue light on repair of UV-B-induced damage. Chin. J. Oceanol. Limnol..

[102] Korbee N., Figueroa F.L., Aguilera J. (2005). Effect of light quality on the accumulation of photosynthetic pigments, proteins and mycosporine-like amino acids in the red alga *Porphyra leucosticta* (Bangiales, Rhodophyta). J. Photochem. Photobiol. B Biol..

[103] Pliego-Cortés H., Bedoux G., Boulho R., Taupin L., Freile-Pelegrín Y., Bourgougnon N., Robledo D. (2019). Stress tolerance and photoadaptation to solar radiation in *Rhodymenia pseudopalmata* (Rhodophyta) through mycosporine-like amino acids, phenolic compounds, and pigments in an Integrated Multi-Trophic Aquaculture system. Algal Res..

[104] Orfanoudaki M., Hartmann A., Karsten U., Ganzera M. (2019). Chemical profiling of mycosporine-like amino acids in twenty-three red algal species. J. Phycol..

[105] Jin N.N., Zhang Z.H., Li B.F. (2012). The constitutes and extraction analysis of mycos-porine-like amino acids (MAAs) in the Gracilariaceae. Mar. Sci..

[106] Volkmann M., Gorbushina A.A. (2006). A broadly applicable method for extraction and characterization of mycosporines and mycosporine-like amino acids of terrestrial, marine and fresh water origin. FEMS Microbiol. Lett..

[107] Zheng Y., Gao K. (2009). Impacts of solar UV radiation on the photosynthesis, growth, and UV-absorbing compounds in *Gracilaria lemaneiformis* (Rhodophyta) grown at different nitrate concentrations. J. Phycol..

[108] Jin N.N. (2012). Study on the Isolation, Purification and Application of Mycosporine-Like Amino Acids (MAAs) in *Gracilaria changii*. Master’s Thesis.

[109] Navarro N.P. (2015). Sunscreens of red algae from Patagonia: A biotechnological perspective. Pure Appl. Chem..

[110] Roleda M.Y., Nyberg C.D., Wulff A. (2012). UVR defense mechanisms in eurytopic and invasive *Gracilaria vermiculophylla* (Gracilariales, Rhodophyta). Physiol. Plant..

[111] Gacesa R., Lawrence K.P., Georgakopoulos N.D., Yabe K., Dunlap W.C., Barlow D.J., Wells G., Young A.R., Lon P.F. (2018). The mycosporine-like amino acids porphyra-334 and shinorine are antioxidants and direct antagonists of Keap1-Nrf2 binding. Biochimie.

[112] Navarro N.P., Figueroa F.L., Korbee N., Mansilla A., Matsuhiro B., Barahona T., Plastino E.M. (2014). The Effects of NO_3_^−^ Supply on *Mazzaella laminarioides* (Rhodophyta, Gigartinales) from Southern Chile. Photochem. Photobiol..

[113] Jiang H.X., Gao K.S., Helbling E.W. (2008). UV-absorbing compounds in *Porphyra haitanensis* (Rhodophyta) with special reference to effects of desiccation. J. Appl. Phycol..

[114] Velasco-Charpentier C., Pizarro-Mora F., Navarro N.P. (2016). Variation in mycosporine-like amino acids concentrations in seaweeds from Valparaiso and Magellan Regions, Chile. Rev. Biol. Mar. Oceanogr..

[115] Karsten U., Hoyer K. (2004). UV-absorbing mycosporine-like amino acids in marine macroalgae and their role in UV protection. Ber. Polarforsch. Meeresforsch..

[116] Hoyer K., Sabine S., Karsten U., Wiencke C. (2003). Interactive effects of temperature and radiation on the mycosporine-like arnino acid contents in polar macroalgae. Ber. Polarforsch. Meeresforsch..

[117] Karsten U., Wiencke C. (1999). Factors controlling the formation of UV-absorbing mycosporine-like amino acids in the marine red alga *Palmaria palmata* from Spitsbergen (Norway). J. Plant Physiol..

[118] Wada N., Sakamoto T., Matsugo S. (2015). Mycosporine-like amino acids and their derivatives as natural antioxidants. Antioxidants.

[119] Pandey A., Pandey S., Rajneesh J.P., Ahmed H., Vidya Singh Shailendra P.S., Sinha R.P. (2017). Mycosporine-like amino acids (MAAs) profile of two marine red macroalgae, *Gelidium* sp. and *Ceramium* sp.. Int. J. Appl. Sci. Biotechnol..

[120] Bhatia S., Sharma K., Sharma A., Purohit A.P. (2011). Mycosporine and mycosporine-like amino acids: A paramount tool against ultra violet irradiation. Pharmacogn. Rev..

[121] Álvarez-Gómez F., Korbee N., Casas-Arrojo V., Abdala-Díaz R.T., Figueroa F.L. (2019). UV photoprotection, cytotoxicity and immunology capacity of red algae extracts. Molecules.

[122] Ryu J., Park S.J., Kim I.H., Choi Y.H., Nam T.J. (2014). Protective effect of porphyra-334 on UVA-induced photoaging in human skin fibroblasts. Int. J. Mol. Med..

[123] Kulkarni A., Lee J.H., Seo H.H., Kim H.S., Cho M.J., Shin D.S., Kim T., Moh S.H. (2015). Photoinduced conductivity in mycosporine-like amino acids. Mater. Chem. Phys..

[124] Ying R., Zhang Z.H., Song W.S., Li B.F., Hou H. (2019). Protective effect of MAAs extracted from *Porphyra tenera* against UV irradiation-dinduced photoaging in mouse skin. J. Photochem. Photobiol. B Biol..

[125] Navarro N.P., Figueroa F., Korbee N., Mansilla A., Jofre J., Plastino E.M. (2016). Differential responses of tetrasporophytes and gametophytes of *Mazzaella laminarioides* (Gigartinales, Rhodophyta) under solar UV radiation. J. Phycol..

[126] Navarr N.P., Figueroa F.L., Korbee N. (2017). Mycosporine-like amino acids vs carrageenan yield in *Mazzaella laminarioides* (Gigartinales; Rhodophyta) under high and low UV solar irradiance. Phycologia.

[127] Ni M.Y. (2014). The study on the Isolation, Purification, Identification and Antioxidant Activity of Mycosporine-Like Amino Acids (MAAs) in *Eucheuma*. Master’s Thesis.

[128] Zhang M.M. (2015). The Preparation Techniques of Mycosporine-Like Amino Acid from *Porphyra yezoensis*. Master’s Thesis.

[129] De la Coba Francisca A.J., Korbee N., María Victoria de Gálvez H.-C.E., Álvarez-Gómez F., Figueroa F.L. (2019). UVA and UVB photoprotective capabilities of topical formulations containing mycosporine-like amino acids (MAAs) through different biological effective protection factors (BEPFs). Mar. Drugs.

[130] Jin N.N., Zhang Z.H., Li B.F., Yan F.F., Sun J.S. (2011). Study on the isolation, purification and composition analysis of mycosporine-like amino acids (MAAs) in *Gracilaria changii*. J. Fish. China.

[131] Hartmann A., Gostner J., Fuchs J.E., Chaita E., Aligiannis N., Skaltsounis L., Ganzera M. (2015). Inhibition of collagenase by mycosporine-like amino acids from marine sources. Planta Med..

[132] Zhang Z.H., Tashiro Y., Matsukawa S., Ogawa H. (2005). Influence of pH and temperature on the ultraviolet-absorbing properties of porphyra-334. Fish. Sci..

[133] Niu M.Y., Zhang Z.H., Gao M., Bu L., Zhang M.M. (2014). Optimization the extraction process of mycosporine-like amino acids from Eucheuma. Acad. Period. Farm Prod. Process..

[134] He Q.M. (2008). Study on Preparation and Character of UV-Absorbing Compound in Seaweed. Master’s Thesis.

[135] Ying R., Zhang Z.H., Duan X.S., Zhao X., Hou H., Li B.F. (2017). Optimization of purification process of mycosporine-like amino acid from *Porphyra haitanensis* and study on its antiultraviolet activity. Mar. Sci..

[136] Tartarotti B., Sommaruga R. (2002). The effect of different methanol concentrations and temperatures on the extraction of mycosporine-like amino acids (MAAs) in algae and zooplankton. Arch. Hydrobiol..

